# Remolding the tumor microenvironment by bacteria augments adoptive T cell therapy in advanced-stage solid tumors

**DOI:** 10.1038/s41392-024-02028-3

**Published:** 2024-11-22

**Authors:** Chaojie Zhu, Chao Liu, Qing Wu, Tao Sheng, Ruyi Zhou, En Ren, Ruizhe Zhang, Zhengjie Zhao, Jiaqi Shi, Xinyuan Shen, Zhongquan Sun, Zhengwei Mao, Kaixin He, Lingxiao Zhang, Yuan Ding, Zhen Gu, Weilin Wang, Hongjun Li

**Affiliations:** 1 Department of Hepatobiliary and Pancreatic Surgery the Second Affiliated Hospital, School of Medicine, Zhejiang University, 310009 Hangzhou, China; 2 National Key Laboratory of Advanced Drug Delivery and Release Systems, College of Pharmaceutical Sciences, Zhejiang University, 310058 Hangzhou, China; 3 Liangzhu Laboratory, Zhejiang University Medical Center, 311121 Hangzhou, China; 4 State Key Laboratory of Stress Biology and Fujian Provincial Key Laboratory of Innovative Drug Target Research, School of Pharmaceutical Sciences, Xiamen University, 361102 Xiamen, China; 5 MOE Key Laboratory of Macromolecular Synthesis and Functionalization, Department of Polymer Science and Engineering, Zhejiang University, 310027 Hangzhou, China; 6 Interdisciplinary Nanoscience Center, Aarhus University, Aarhus C, DK-8000 Denmark; 7 Key Laboratory of Precision Diagnosis and Treatment for Hepatobiliary and Pancreatic Tumor of Zhejiang Province, 310009 Hangzhou, China; 8 ZJU-Pujian Research & Development Center of Medical Artificial Intelligence for Hepatobiliary and Pancreatic Disease, 310058 Hangzhou, China; 9 Jinhua Institute of Zhejiang University, 321299 Jinhua, China; 10 Department of General Surgery, Sir Run Run Shaw Hospital, School of Medicine, Zhejiang University, 310016 Hangzhou, China

**Keywords:** Tumour immunology, Cell delivery

## Abstract

The intricate tumor microenvironment presents formidable obstacles to the efficacy of adoptive T cell therapy in the management of solid tumors by limiting the infiltration and inducing exhaustion of the transferred T cells. Here, we developed a bacterial-based adjuvant approach that augments the efficacy of adoptive T-cell therapy for solid tumor treatment. Our study reveals that intratumor injection of *E. coli* MG1655 normalizes tumor vasculatures and reprograms tumor-associated macrophages into M1 phenotype that produce abundant CCL5, together facilitating tumor infiltration of adoptively transferred T cells. The depletion of tumor-associated macrophages or CCL5 neutralization in vivo leads to the significantly decreased solid tumor infiltration of adoptive T cells in the presence of bacteriotherapy. This combinatorial therapy, consisting of *E. coli* adjuvant and adoptive T-cell therapy, effectively eradicates early-stage melanoma and inhibits the progression of pancreatic tumors. Notably, this dual strategy also strengthened the distal tumor control capabilities of adoptive T-cell therapy through the induction of in situ tumor vaccination. This dual therapeutic approach involving bacterial therapy targeting the interior of solid tumors and adoptive T-cell therapy attacking the tumor periphery exhibits potent therapeutic efficacy in achieving the eradication of advanced-stage tumors, including melanoma and hepatocellular carcinoma, by converging attacks from both inside and outside the tumor tissues.

## Introduction

The intratumor microbiota has emerged as a focal point of intense research due to its multifaceted interactions with host tumors, revealing a complex interplay that significantly impacts tumor progression and treatment outcomes. Recent advancements in this field have underscored the importance of understanding the intricate relationships between intratumor microbiota and their hosts, as these interactions can elicit both pro-tumorigenic and therapeutic effects.^1,2^ On one hand, intratumor microbiota have been implicated in promoting tumorigenesis through various mechanisms. They can induce mutations in tumor cells, thereby fostering the development of more aggressive cancer phenotypes.^3^ Chronic inflammation, another hallmark of cancer, is also fueled by intratumor microbiota, creating an environment conducive to tumor growth and progression.^4^ Moreover, these microbiota can diminish the efficacy of therapeutic drugs by altering the tumor microenvironment and promoting drug resistance.^5^ Additionally, they play a pivotal role in facilitating the formation of metastatic niches, which enable cancer cells to spread and colonize distant organs.^6^

Despite these defects for cancer treatment, it is equally important to recognize their therapeutic potential. Tumor microbiota possess unique properties that can be harnessed in favor of antitumor responses. For instance, their ability to target hypoxic regions within tumors offers a promising strategy for improving the delivery and efficacy of therapeutic agents.^7^ In addition to hypoxic targeting, intratumor microbiota can activate innate immune sensing pathways.^8,9^ This activation can stimulate the production of cytokines and chemokines, which can recruit and activate immune cells to attack the tumor. Furthermore, intratumor microbiota can modulate host metabolism, altering the availability of nutrients and metabolites that support tumor growth. By manipulating these metabolic pathways, intratumor microbiota can be harnessed to create an environment that is hostile to cancer cells.^10^ Intratumor microbiota can also induce the formation of tertiary lymphoid structures (TLSs) within the tumor microenvironment.^11^ And the presence of TLSs has been associated with better treatment outcomes in various cancer types. These inherent features further shed light on their possibilities in remolding the tumor microenvironment to favor antitumor responses.^12–14^

In this study, we discovered that intratumor injection of *E. coli* MG1655 has the potential to serve as a versatile adjuvant therapeutic approach to enhance the efficacy of adoptive T cell transfer. Adoptive T cell therapy, particularly chimeric antigen receptor (CAR)-T cell therapy, has emerged as a revolutionary approach in the treatment of hematological malignancies, achieving notable successes in clinical trials and real-world applications.^15–17^ However, the effectiveness of CAR-T therapy in solid tumors remains a significant challenge.^18,19^ Solid tumors are distinguished by abnormal vascularization, a dense stromal matrix that impedes the infiltration of cytotoxic T cells, and a robust immunosuppressive tumor microenvironment that promotes T cell exhaustion.^20–22^ In contrast to hematological cancers, the successful application of adoptive T cell therapy in solid tumors necessitates sophisticated genetic manipulation and engineering of T cells to counteract the suppressive tumor milieu.^23,24^ Alternatively, harnessing the innate immune-stimulatory properties of bacteria, particularly the surface molecules such as lipopolysaccharides, flagellin, and peptidoglycan, offers a promising alternative strategy to ignite the tumor microenvironment and amplify the therapeutic response of adoptively transferred T cells.^25,26^

**Fig. 1:**
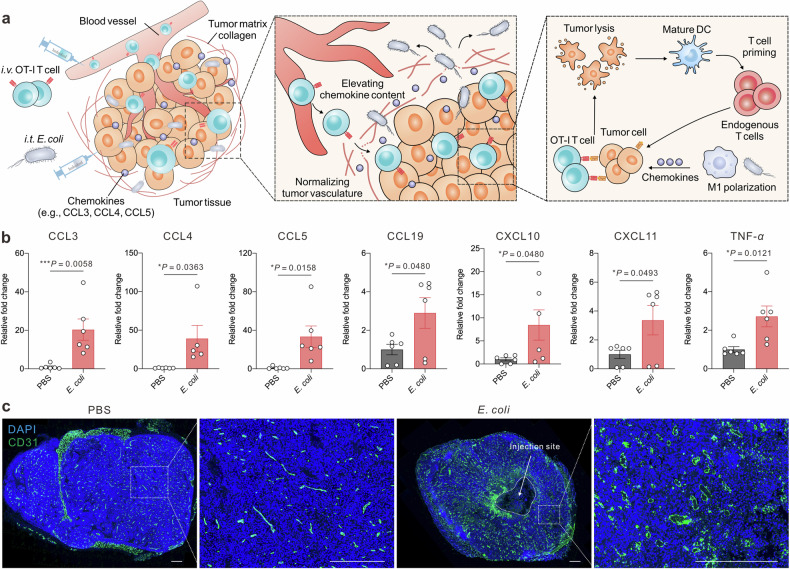
Intratumor bacteria therapy remolds tumor physical and physiochemical microenvironment. **a** Schematic illustration of the bacterial therapy in adjuvating adoptive T cell therapy. *E. coli* elevates intratumor T cell-chemotaxis chemokines, normalizes tumor vasculature and remolds tumor microenvironments to promote T cells’ entry and killing efficiency. **b** The relative content of intratumor T cell-centered cytokine and chemokines in the control and *E. coli*-treated groups. Data are shown as mean ± s.e.m., *n* = 5–6 biologically independent samples. *P* values were determined by unpaired, two-tailed *t*-test. **c** Representative fluorescence staining of intratumor CD31, scale bar = 500 μm

Intratumor bacterial therapy could modulate the physical and chemical cues of tumor tissues, including normalizing tumor vasculatures and elevating the concentration of a series of T-cell centered chemokines including CCL3, CCL4, and CCL5 (Fig. 1a).^27–30^ These chemokines play crucial roles in recruiting and activating immune cells within the tumor microenvironment. In particular, we discovered that *E. coli* MG1655 reprogrammed tumor-associated macrophages into the M1 phenotype, which secreted CCL5 to recruit the adoptively transferred T cells. By augmenting the tumor accumulation of cytotoxic T cells, this combinatorial therapeutic strategy achieved 87.5% tumor remission rates in small murine melanomas and showed superior therapeutic efficacy against subcutaneous pancreatic tumors. Furthermore, the immunostimulatory properties of *E. coli*, together with adoptive transferred T cells, launched in situ tumor vaccinations through augmenting the tumor neoantigen presentation and maturation of intratumor dendritic cells and priming of circulating CD8 T cells for distal tumor control. Spatially orchestrated, intratumor *E. coli* MG1655 and adoptive T cells infiltrating the tumor periphery display a synergistic potential to achieve complete remission in advanced-stage solid tumors. This localized interplay harnessed the unique properties of both modalities, fostering a robust antitumor response tailored to the complex tumor microenvironment.

## Results

### Intratumor *E. coli* modulates tumor physical and physiochemical microenvironment

We first determined the cytokine and chemokine profile in B16F10-OVA tumors following intratumor (i.t.) injection of *E. coli*. Luminex assay showed an elevation in TNF-*α* and nearly all chemokines, with special notice to CCL3, CCL4, CCL5, CCL19, CXCL10, and CXCL11, which were reported as crucial CD8 T cell recruiting chemokines (Fig. 1b and Supplementary Fig. 1). We observed 19.4-fold, 38.1-fold, 32.4-fold, 1.89-fold, 7.44-fold, and 2.36-fold increments of CCL3, CCL4, CCL5, CCL19, CXCL10, and CXCL11, respectively, in the *E. coli*-treated group. We then explored the impact of *E. coli* on the physical levels of tumor tissues. Tumor vasculatures have been reported to inhibit T cell extravasation, suppress T cell activities, and mediate FasL-induced T cell apoptosis.^31^ The normalization of tumor vessels could assist in the infiltration of cytotoxic T cells. We stained tumor vasculatures with CD31 and observed intratumor *E. coli* could destroy tumor vasculatures at the injection site and augment the formation and normalization of tumor vasculature in the periphery, which could facilitate the extravasation of circulating T cells into tumor tissues (Fig. 1c and Supplementary Fig. 2).^30,32^

### Intratumor *E*. *coli* treatment augments OT-I T cell infiltration into solid tumors

**Fig. 2:**
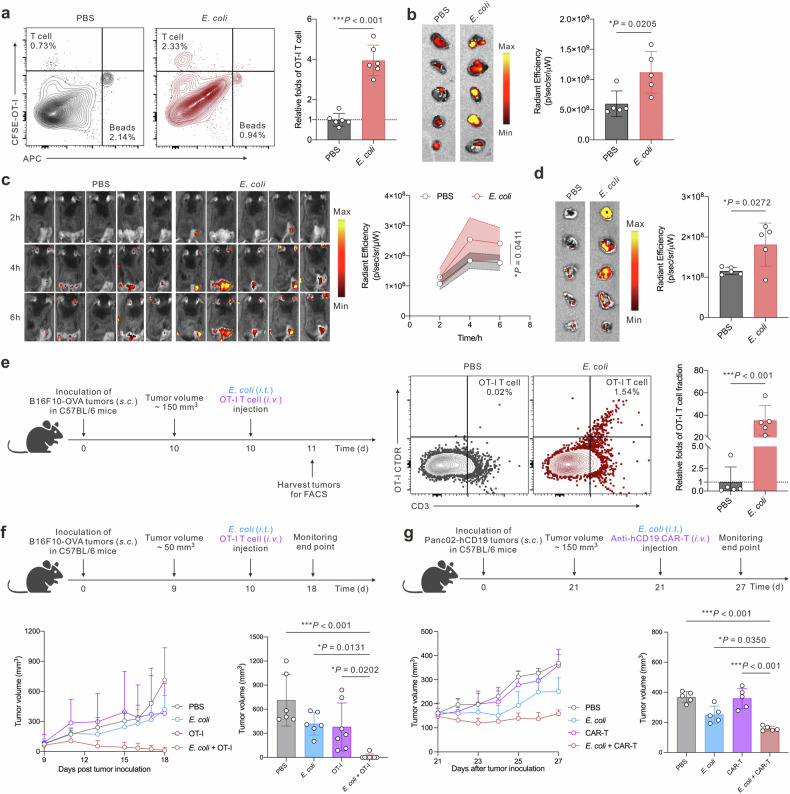
Intratumor *E. coli* therapy promotes OT-I T cell infiltration for augmented tumor control. **a** Transwell migration assay of OT-I T cells (CFSE-labeled). Representative flow cytometry graphs, and statistical analysis of T cell infiltration relative folds (calculated by bottom well-located OT-I T cells to counting beads ratio, *n* = 6 biologically independent samples). **b** Diagram and analysis of the tumor and OT-I T cell (labeled by Cy5) co-culture recruitment assay (*n* = 5 biologically independent samples). **c** Serial IVIS images and statistical quantification of Cy5-T cell signals in tumor-bearing mice (*n* = 5 biologically independent samples). **d** IVIS images and statistical quantification of Cy5 signals in the harvested tumors post 24-h injection of OT-I T cells (*n* = 5 biologically independent samples). **e** Representative flow cytometry graphs of tumor-infiltrating OT-I T cells and statistical analysis of intratumor OT-I T cell fraction in tumor tissues (*n* = 5 biologically independent animals). **f** Therapeutic schedule and tumor volume monitoring of the combination therapy of *E. coli* with OT-I T cells for subcutaneous murine B16F10-OVA treatment (*n* = 6–8 biologically independent animals). **g** Therapeutic schedule and tumor volume monitoring of the combination therapy of *E. coli* with murine anti-hCD19 CAR-T cells for subcutaneous murine Panc02-hCD19 tumor treatment (*n* = 5 biologically independent animals). Data are shown as mean ± s.d. *P* values were determined by unpaired, two-tailed *t*-test for **a**–**e** and one-way ANOVA with a Tukey post hoc test for (**f**) and (**g**)

As *E. coli* elevated intratumor T cell-centered chemokines, we preliminarily tested whether the tumor interstitial fluid could directly promote T cell infiltration. We derived OT-I T cells as a model for tumor-reactive T cells and assessed the CD8 T cell population along with their tumor-killing efficacy (Supplementary Fig. 3). We placed B16F10-OVA cells and tumor fluid content at the bottom well with the upper well loaded with CFSE-labeled OT-I T cells. The flowcytometric analysis showed a higher OT-I T cells to beads ratio in the *E. coli* group, ~3.95 folds of that in the PBS group (Fig. 2a). In vivo imaging system (IVIS) image also revealed a 0.52-fold elevation of CFSE intensity in the *E. coli* group (Supplementary Fig. 4). To further validate the T cell recruiting efficacy of the elevated intratumor chemokine profiles induced by *E. coli*, we tested on whole tumor tissue. The resected whole B16F10-OVA tumor tissues with pretreatment of i.t. PBS or i.t*. E. coli* were co-cultured with OT-I T cells. IVIS image determined a higher Cy5 signal of OT-I T cells (~1.87 folds of the control group) in the *E. coli* group (Fig. 2b). To ascertain whether intratumor *E. coli* treatment could directly promote OT-I T cell infiltration into the tumor tissues, we repeated the co-culture assay as Fig. 2b, with whole tumor tissues substituted by surgical-derived half tumors. In comparison with the whole tumor setting, the inner tissue of the half tumors could directly interface with OT-I T cells. As a result, a higher Cy5.5 signal of OT-I T cells was revealed in the *E. coli* group, about 1.21-fold of that in the control group (Supplementary Fig. 5).

Based on the above in vitro evidence, we then determined the tumor infiltration condition of OT-I T cells in vivo. OT-I T cells were pre-labeled with Cy5 and intravenously injected (i.v.) after i.t. PBS or i.t*. E. coli*. The IVIS image revealed that OT-I CD8 T cells infiltrated into tumor tissues at a higher quantity in the *E. coli* group (Fig. 2c). Tumors were harvested and quantified for Cy5 intensities after 24 h post-treatment. The Cy5 intensities in the *E. coli* group were nearly 1.57-fold of that in the PBS group (Fig. 2d). We leveraged flow cytometry to quantify the infiltration increment precisely. As a result, the proportion of the infiltration OT-I T cell fraction of tumor tissues in the *E. coli* group was ~35.3-fold of that in the PBS group (Fig. 2e). We initially tested whether the improved tumor accumulation of tumor-specific T cells could bring therapeutic advantages. We adopted OT-I T cells and anti-hCD19 murine CAR-T cells for B16F10-OVA and Panc02-hCD19-luci tumor treatment, respectively (Supplementary Fig. 6).^33,34^ As a result, this combination achieved 87.5% complete tumor remission in melanoma and significantly inhibited pancreatic tumor growth (Fig. 2f, g). Importantly, the mice’s body weight was kept in the normal range in both experiments (Supplementary Fig. 7). In addition, we also monitored the long-term survival of this combination strategy for murine melanoma treatment. *E. coli* + OT-I group achieved a 6/7 survival rate up to 40 days (Supplementary Fig. 8). Routine blood tests, serological analysis, and H&E images showed no significant variations compared to the healthy mice at the same age (Supplementary Figs. 9, 10).

### *E. coli* MG1655 reprograms TAM to secrete CCL5 for recruiting the adoptive transferred T cells

We further delved into elucidating the specific mechanisms underpinning the enhancement of T cell tumor infiltration efficacy mediated by intratumor bacterial therapy. The initial response of the endogenous innate immune system to exogenous bacterial invasion holds paramount importance in this process.^35^ During this intricate interplay, several crucial chemokines, including CCL3, CCL4, and notably CCL5, have been identified as key players in orchestrating T cell recruitment towards the tumor microenvironment.^36^ Drawing upon the comprehensive data gathered from Luminex assay analysis (Fig. 1b and Supplementary Fig. 1), we decided to focus our investigations on the CCL5 signaling axis. This choice was predicated on the significance of CCL5 in directing T cell migration and its potential to serve as a pivotal mediator in augmenting the efficacy of intratumor bacterial therapy.^37^

**Fig. 3:**
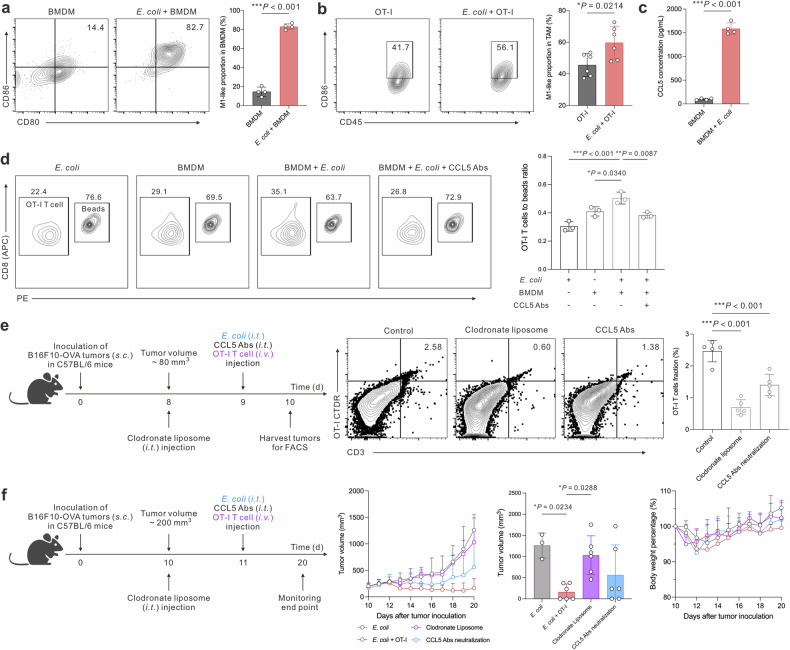
*E. coli* reprograms TAM to secrete CCL5 for recruiting the adoptively transferred T cells. **a** Representative flow cytometry graphs and statistical analysis of M1 proportion in BMDM with different treatments. **b** Representative flow cytometry graphs and statistical analysis of M1 proportion of TAM in B16F10-OVA tumors with different treatments. **c** ELISA assay for determining CCL5 secretion capability of BMDM and B16F10-OVA tumor cells with or without co-culture with *E. coli*. **d** Representative flow cytometry graphs and statistical analysis of transmigration OT-I CD8 T cell quantity over counting beads. OT-I CD8 T cells were located at the upper well, and different cell supernatants were placed at the bottom well. **e** Formulation injection schedule and representative flow cytometry graphs of intratumor infiltrated OT-I T cell proportions (*n* = 5 biologically independent animals). **f** Therapeutic schedule of TAM depletion or CCL5 neutralization, statistic monitoring of tumor volume and body weight percentage (*n* = 3–6 biologically independent animals). Data are shown as mean ± s.d. *P* values were determined by unpaired, two-tailed *t*-test for **a**–**c** and one-way ANOVA with a Tukey post hoc test for (**d**)–(**f**)

We initially conducted co-culture experiments between bacteria and bone marrow-derived macrophages (BMDM), discovering that *E. coli* MG1655 significantly upregulated the expression of M1-phenotype markers in BMDM (Fig. 3a). This finding suggests a shift towards an inflammatory macrophage phenotype induced by *E. coli*. Furthermore, in the context of tumor-associated macrophages, we observed an elevated level of CD86 expression in the presence of *E. coli* within the OT-I group, compared to the OT-I group alone (Fig. 3b). Moreover, the addition of *E. coli* notably boosted BMDM to secrete CCL5 (Fig. 3c).^38^ To understand the potential role of CCL5 in this setting, we analyzed the expression of the receptor of CCL5, CCR5, on OT-I CD8 T cells using flow cytometry (Supplementary Fig. 11). For functionally assessing the impact of *E. coli*-induced CCL5 on T cell infiltration, we employed a transwell migration assay.^39^ Our results demonstrated that the supernatant of the coculture (*E. coli* with BMDM) led to the highest level of OT-I CD8 T cell transmigration (Fig. 3d). Furthermore, the ability to block T cell infiltration through the use of the CCL5 neutralization antibody underscores the critical role of CCL5 in directing the migration of OT-I CD8 T cells towards the tumor site.

Furthermore, we explored the impact of the tumor-associated macrophage (TAM) depletion agent, clodronate liposome, and CCL5 neutralization antibody, on the accumulation of OT-I CD8 T cells within the tumor tissues (Fig. 3e). Our flow cytometry analysis revealed that both treatments significantly reduced the proportion of intratumor OT-I T cells. However, it is noteworthy that the extent of downregulation was less pronounced in the CCL5 antibody group compared to the clodronate liposome group. This observation could potentially be attributed to the presence of additional CCL5-independent chemotaxis players that contribute to T cell infiltration within the tumor.^40^ To assess the therapeutic implications of the reduced OT-I T cell infiltration induced by clodronate liposome or CCL5 neutralization antibody, we conducted an analysis of tumor growth inhibition (Fig. 3f). Notably, the tumor inhibitory effect was substantially diminished in the clodronate liposome group compared to the *E. coli* group, indicating the importance of TAMs in facilitating T cell-mediated tumor suppression. The mean tumor volume at the end of the monitoring time point was the lowest in the *E. coli* + OT-I T cells group, mirroring the critical role of both TAMs and CCL5 in mediating T cell infiltration for potentiating the antitumor immune response.

### *E. coli* adjuvants transferred T cells for distal tumor control through in situ tumor vaccination

**Fig. 4:**
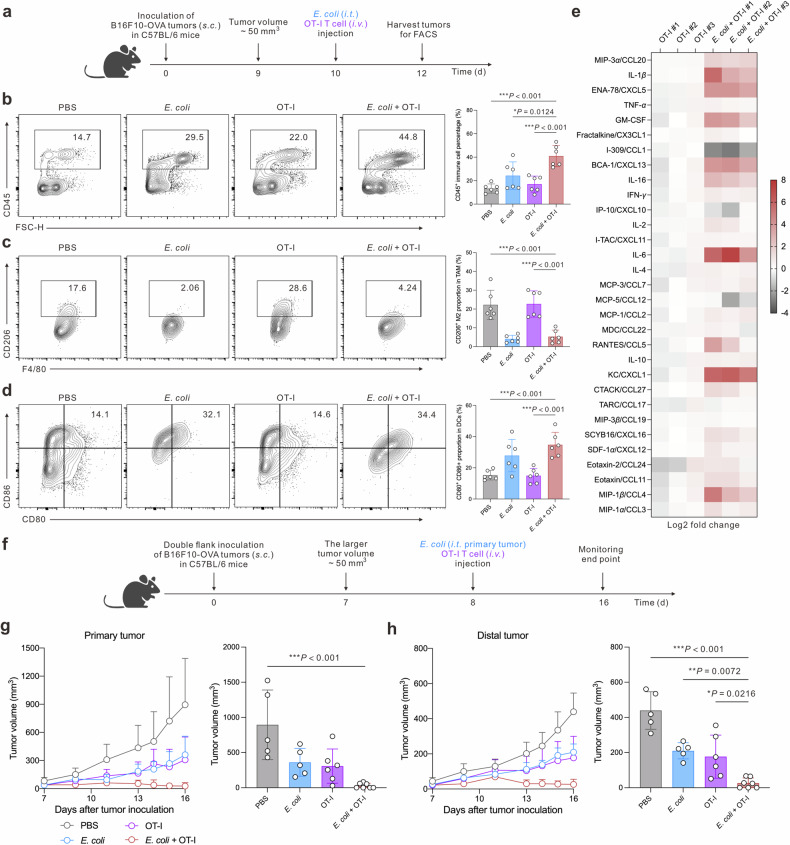
Combination therapy vaccinates tumor in situ for distal tumor control. **a** Therapeutic time schedule for intratumor phenotyping. **b** Representative flow cytometry graphs and statistical analysis of intratumor CD45^+^ immune cell percentage (*n* = 6 biologically independent animals). **c** Representative flow cytometry graphs and statistical analysis of intratumor CD206^+^ macrophage fraction among tumor-resident macrophages (*n* = 6 biologically independent animals). **d** Representative flow cytometry graphs and statistical analysis of intratumor CD80^+^ CD86^+^ matured DC fraction in tumor-resident DCs (*n* = 6 biologically independent animals). **e** Heat map of intratumor cytokine and chemokine in different groups. Data are calculated by log2 fold change in comparison with the average of the OT-I group, *n* = 3 biological independent animals. **f** Therapeutic schedule for double flank murine melanoma treatment. **g** Tumor volume monitoring and statistical analysis of the primary tumors (*n* = 5–7 biologically independent animals). **h** Tumor volume monitoring and statistical analysis of the distal tumors (*n* = 5–7 biologically independent animals). Data are shown as mean ± s.d. *P* values were determined by one-way ANOVA with a Tukey post hoc test

Given that *E. coli* present a diverse array of pathogen-associated molecular patterns, they possess the capability to prime the innate immune system, which in turn can stimulate adaptive immune responses directed against pathogens or, in this context, tumor cells.^41^ To investigate the impact of *E. coli* on intratumor immune cell phenotypes, we conducted flow cytometry analysis (Fig. 4a). Our findings revealed that the combination of *E. coli* and OT-I T cells elicited the highest levels of intratumor immune cell infiltration (Fig. 4b). Notably, both the *E. coli* group and the *E. coli* + OT-I T cell group significantly reduced the proportion of M2-like macrophages (Fig. 4c) and induced the maturation of intratumor dendritic cells, as evidenced by increased expression of CD80 and CD86 (Fig. 4d). Furthermore, to gain insights into the underlying mechanisms mediating the crosstalk between innate and adaptive immunity, we utilized a Luminex assay to quantify intratumor chemokine and cytokine levels. Our results indicated that the addition of *E. coli* successfully reinvigorated the tumor microenvironment compared to OT-I T cells alone, as demonstrated by the upregulation of various chemokines and cytokines essential for immune cell recruitment, activation, and effector function (Fig. 4e).^42,43^ These findings underscore the pivotal role of *E. coli* in orchestrating the crosstalk between innate and adaptive immunity, thereby potentiating antitumor immune responses.

Building upon our previous findings, we extended our investigation to a double-flank melanoma mouse model. This model could partially offer a valuable tool to assess the therapeutic potential of locally administered treatments in influencing tumor growth at distant sites or metastasis, providing insights into their systemic effects and potential for controlling disease progression beyond the primary tumor location. Specifically, when the larger tumor reached approximately 50 mm^3^, we administered *E. coli* and OT-I T cells (Fig. 4f). Our results demonstrated that the *E. coli* + OT-I group exhibited remarkable therapeutic efficacy, achieving tumor eradication in 4 out of 7 in primary tumors and 3 out of 7 in distal tumors (Fig. 4g, h). Notably, we observed that *E. coli* alone was also capable of inhibiting the growth of distal tumors, prompting us to investigate deeper into the mechanisms underlying this distal tumor-restraining effect. To explore these mechanisms, we focused on the potential of *E. coli* to enhance the tumor antigen presentation capabilities of dendritic cells (DCs), which are crucial for priming tumor-specific T cells that can mediate distal tumor control. Flow cytometry analysis revealed that the *E. coli* + OT-I group had the highest proportion of H-2Kb SIINFEKL-presenting DCs (Supplementary Fig. 12a).^44^ This, coupled with the pro-maturation effects of *E. coli* as an adjuvant (Fig. 4d), suggests that *E. coli* can elicit robust endogenous antitumor immune responses. Furthermore, we observed that the activation state of circulating CD8 T cells was significantly enhanced in both the *E. coli* and *E. coli* + OT-I groups (Supplementary Fig. 12b). This finding underscores the ability of *E. coli* to stimulate systemic immune responses that extend beyond the primary tumor site, contributing to the observed distal tumor-restraining effect.

### Combination treatment eradicates advanced-stage melanoma and hepatocellular carcinoma

**Fig. 5:**
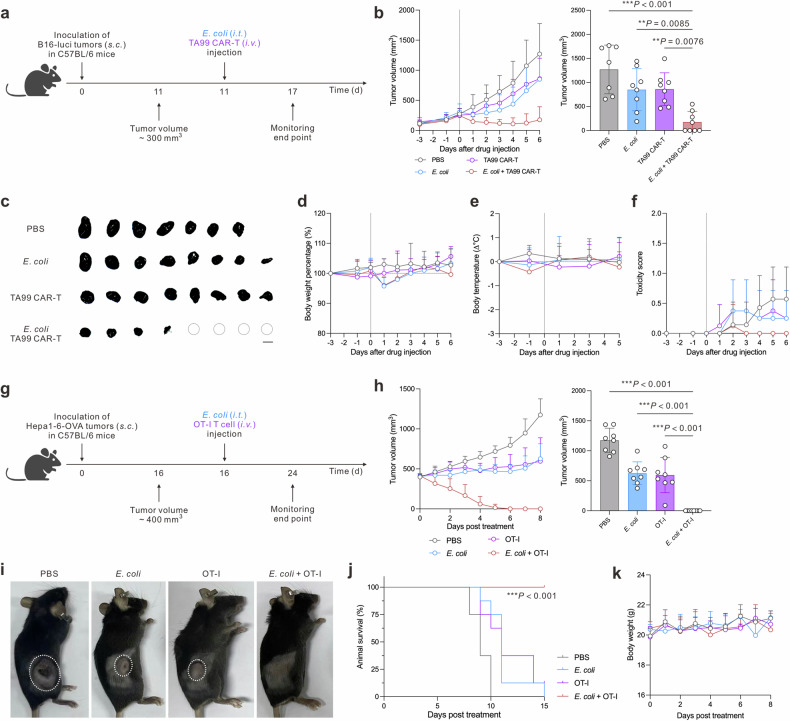
Combination therapy eliminates advanced-stage murine melanoma and hepatocellular carcinoma. **a** Therapeutic schedule for advanced-stage murine melanoma treatment (*n* = 7–8 biologically independent animals). **b** Tumor volume monitoring and statistical analysis for B16-luci tumor model. **c** Photographs of the harvest tumors at the monitoring end timepoint, scale bar = 1 cm. A dotted circle indicates a single mouse in which the tumor was eradicated. Toxicity monitoring of mice in different treatment groups for body weight in **d**, body temperature in **e**, and toxicity score in (**f**). **g** Therapeutic schedule for advanced-stage murine hepatocellular carcinoma treatment (*n* = 8 biologically independent animals). **h** Tumor volume monitoring and statistical analysis of tumors for different treatment groups. **i** Representative tumor-bearing mice post-treatment. **j** Mice survival curve monitoring for different treatment groups. **k** Body weight monitoring for different treatment groups. Data are shown as mean ± s.d. *P* values of final tumor volume were determined by one-way ANOVA with a Tukey post hoc test. The survival statistical significance was analyzed by log-rank (Mantel–Cox) test

Given the promising therapeutic efficacy and notable tumor regression observed in preclinical models of small tumors, we further explored the therapeutic potential of this combined treatment approach in advanced-stage tumors. For this purpose, we generated TA99 murine CAR-T cells that could specifically recognize melanoma-associated tyrosinase-related protein 1 (TRP-1) and rigorously characterized their phenotypic profile (CD4/CD8 ratio and CCR5 expression) as well as their ability to specifically eliminate B16-luci tumor cells in vitro (Supplementary Fig. 13).^45,46^ When applied to murine melanoma models where tumor volumes had reached 300 mm^3^, the combination of *E. coli* with TA99 CAR-T cells resulted in a remarkable 50% complete tumor remission rate (Fig. 5a–c). Furthermore, the therapeutic regimen demonstrated satisfactory tolerability, with no significant adverse effects on mouse body weight or temperature throughout the treatment period (Fig. 5d–f). For the treatment of hepatocellular carcinoma, we applied a combination of *E. coli* and OT-I CD8 T cells once the tumor volume reached 400 mm^3^ (Fig. 5g).^47^ Remarkably, the *E. coli* + OT-I T cell group achieved a tumor-free rate of 100% (Fig. 5h, i). Notably, all mice in this group remained alive for 15 days post-treatment (Fig. 5j). The body weights of mice in the *E. coli* + OT-I T cell group kept within the normal range throughout the treatment period, indicating the satisfactory tolerability of this therapeutic approach (Fig. 5k).

### Spatial cooperation between *E. coli* and transferred T cells eliminates solid tumor

**Fig. 6:**
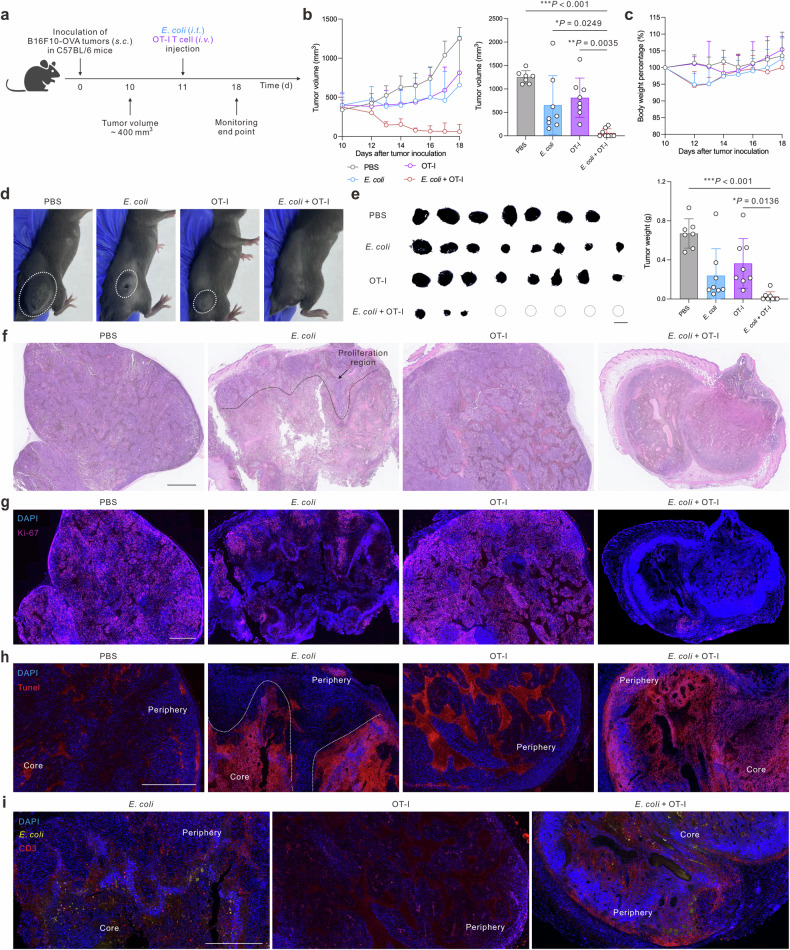
Spatial cooperation of combination therapy eradicates advanced-stage melanoma. **a** Therapeutic schedule of advanced-stage melanoma treatment (*n* = 7–8 biologically independent animals). **b** Tumor growth curves and statistical analysis of tumor volume for different treatment groups. **c** Body weight change curves post-treatment. **d** Representative images of tumor-bearing mice after different treatments. **e** Photographs and tumor weight analysis of the harvested tumors from different treatment groups, scale bar = 1 cm. A dotted circle indicates a single mouse in which the tumor was eradicated. **f** Representative H&E images of tumor sections of different treatment groups, scale bar = 1 mm. **g** Representative fluorescence intratumor staining of Ki-67, scale bar = 1 mm. **h** Representative immunofluorescent intratumor staining of TUNEL, scale bar = 1 mm. **i** Representative intratumor staining of CD3 and *E. coli*, scale bar = 1 mm. Data are shown as mean ± s.d. *P* values were determined by one-way ANOVA with a Tukey post hoc test

Given the exceptional anti-tumor efficacy demonstrated in advanced-stage solid tumors, we started an investigation to elucidate the underlying mechanisms of this therapeutic strategy. To this end, we initiated the treatment modalities once the tumors had grown to 400 mm^3^ (Fig. 6a). As a result, the tumor volume in the combinatory treatment group (*E. coli* + OT-I) decreased significantly, in stark contrast to the continued growth observed in all other treatment groups (Fig. 6b). Importantly, the body weights of the mice remained stable and within the normal range throughout the treatment period, indicating good tolerability (Fig. 6c). Notably, five out of eight mice in the *E. coli* + OT-I group achieved a tumor-free state, further underscoring the potency of this combined therapeutic approach (Fig. 6d, e). To gain a deeper understanding of the mechanisms underlying the remarkable therapeutic efficacy observed, we performed histological and immunohistochemical analyses of the tumors using hematoxylin and eosin (H&E) staining, Ki-67 proliferation marker, and terminal deoxynucleotidyl transferase dUTP nick end labeling (TUNEL) assay. Specifically, we observed a necrotic area in the core of tumors treated with *E. coli* alone, the tumor periphery in the OT-I group, and a nearly complete region in the *E. coli* + OT-I combinatory group (Fig. 6f).^48^

Further analysis with Ki-67 and TUNEL staining revealed a reversed location signature, with decreased proliferation (Ki-67) and increased apoptosis (TUNEL) in the *E. coli* + OT-I group, mirroring the trends observed in the H&E images (Fig. 6g, h). These findings are consistent with previous evidence suggesting that intratumor bacterial therapy can delay tumor growth but may be insufficient to control the rapid outgrowth of the tumor periphery.^49^ However, our results demonstrate that the addition of OT-I CD8 T cells to the *E. coli* therapy significantly enhances its efficacy, resulting in a more comprehensive and effective tumor regression. Based on our findings, we hypothesize that intratumor administration of *E. coli* is capable of effectively destroying the hypoxic inner core of solid tumors but may be limited in its ability to restrain tumor growth at the periphery. In contrast, adoptive T cell therapy exerts a more localized, albeit less potent, therapeutic effect primarily focused on the tumor periphery.^50^ The combination of these two therapies, however, not only directly targets and destroys the inner tumor core but also significantly enhances the infiltration and tumor killing of T cells within the tumor periphery. To strengthen our hypothesis, we located intratumor T cells and *E. coli* (Fig. 6i). *E. coli* was discovered to colonize primarily within the hypoxic inner core of the tumor. In the OT-I group, T cells were localized primarily at the tumor periphery.^51^ However, in the combination therapy group, we observed a significantly higher quantity of T cells infiltrating the tumor tissues, ultimately leading to complete control of advanced-stage solid tumor growth throughout the entire tumor area. These findings further support our hypothesis that the combination of intratumor *E. coli* and adoptive T cell therapy offers a synergistic and potent approach for the treatment of advanced-stage solid tumors.

### Intratumor bacterial therapy promises therapeutic biosafety

One significant concern regarding the use of bacteria in therapeutic applications is the potential for toxicity stemming from bacterial infections.^52^ To ensure the safety and efficacy of bacterial therapy, it is crucial to achieve successful colonization of bacteria within tumor tissues, which necessitates careful optimization and testing of the bacterial dosage. In our study, we employed LuxCDABE-transduced *E. coli* MG1655 to monitor the colonization of intratumor *E. coli*. Our results indicated that the bioluminescence of *E. coli* declined rapidly within the initial 12 h and then remained stable for at least five days (Supplementary Fig. 14). On the endpoint day, we confirmed that the majority of *E. coli* were confined to the tumor tissues, with minimal impact on other major organs (Supplementary Fig. 15). To assess the potential for bacterial infection to induce a cytokine storm, we monitored both intratumor and serum cytokine levels throughout the therapeutic procedure. Notably, all inflammatory cytokine markers, including IFN-*γ*, TNF-*α*, IL-1*β*, IL-6, and IL-10, exhibited a transient increase in serum samples immediately after the initial bacterial injection. However, these levels rapidly returned to normal ranges, similar to pre-injection levels (Supplementary Fig. 16). This suggests that our bacterial therapy was well-tolerated and did not induce severe systemic inflammation, highlighting the potential for safe and effective bacterial-based therapies in the treatment of tumors. We further performed routine blood and serological tests two days post-treatment. The white blood cell, lymphocyte, neutrophil, and red blood cell were all in the normal range (Supplementary Fig. 17). The neutrophil exhibited higher in the *E. coli* and the *E. coli* + OT-I groups.^53^ No significant change was observed in creatinine (CRE-J), total bilirubin (TBIL), and urea (Supplementary Fig. 18). All the treatment groups except the PBS group effectively downregulated alanine aminotransferase (ALT) and aspartate aminotransferase (AST) concentration.

## Discussion

In this study, we discovered that injection of the pristine *E. coli* MG1655 strain could inhibit both primary and distal tumors. This finding adds to the growing body of evidence supporting the use of bacteria as agents for cancer treatment, a field that has a long history dating back to 1891 and has been extensively studied in recent decades.^54^ Public discourse and concerns on bacteriotherapy predominantly focus on its biosafety, specifically the potential risks associated with bacterial infections. These concerns are understandable, given the infectious nature of bacteria.^55^ In the present investigation, we selected the *E. coli* MG1655 strain due to its prevalent employment in molecular research and its sensitivity toward antibiotics for safety control. This strain has been characterized as non-pathogenic through laboratory investigations, which provides some reassurance regarding its safety.^56,57^ Despite the non-pathogenic characterization of *E. coli* MG1655, rigorous security and long-term safety examination by authoritative regulatory entities remains imperative. Safety is paramount in any medical treatment, and it is crucial to ensure that the benefits of bacteriotherapy outweigh the risks. Therefore, additional preclinical and clinical studies are needed to fully evaluate the safety and efficacy of *E. coli* MG1655 and other bacterial strains used in cancer treatment. Other microbes, such as Bacillus Calmette-Guérin (BCG) vaccine^58^ and *Salmonella* VNP20009,^59^ have been tested clinically, which renders feasible subjects to combine with current trial-ongoing CAR-T products for solid tumor clinical tests.

Regarding clinical translation, the rapid proliferative nature of bacteria offers a significant cost advantage compared to contemporary immunotherapeutic approaches. This is particularly beneficial in the context of cancer treatment, where the high cost of therapies can be a prohibitive factor for many patients. Bacteria’s intrinsic tumor-targeting properties allow for localized therapeutic action within the tumor microenvironment, reducing the potential for systemic toxicity and enhancing the efficacy of treatment. With the rapid advancements in synthetic biology, there is immense potential to develop functional bacteria that can respond to environmental cues and produce bioactive therapeutic proteins as adjuvant therapies for augmented adoptive T cell therapy.^60^ These engineered bacteria could potentially enhance the antitumor immune response by delivering cytokines, chemokines, or other immune-modulating factors directly to the tumor site. However, as a form of living therapy, bacterial modalities encounter similar challenges to adoptive T cell therapies, such as ensuring consistent cell viability and anticancer performance of these living biologics. Ensuring the quality and safety of bacterial cultures is critical for large-scale production. This requires the establishment of robust manufacturing processes that can maintain consistent and viable bacterial cultures while simultaneously conducting rigorous evaluations to detect any deleterious genetic alterations or contamination. Furthermore, it is crucial to address safety concerns related to gene stability and potential horizontal gene transfer between the therapeutic bacteria and the microbiome within the human body. Horizontal gene transfer could lead to unintended genetic alterations in the microbiome, potentially causing adverse effects. Therefore, thorough research is needed to understand the genetic stability of therapeutic bacteria.^55^

Our study provides fresh insights into the mechanisms by which bacteria can alter the tumor microenvironment, particularly by sensitizing stromal components and enhancing T cell infiltration into solid tumors. By entering tumor tissue, bacteria have the ability to shift macrophage polarization towards the M1 type and promote the maturation of dendritic cells. This transformation primes the tumor microenvironment, making it more favorable for T cell infiltration and activation. To explore the feasibility and potential efficacy of this approach, we conducted preliminary evaluations across murine models of melanoma, hepatocellular carcinoma, and pancreatic cancer. Our findings suggest that this strategy holds promise for improving the response to immunotherapy in these tumor types, especially suitable for the future application of those that are challenging to treat with surgical resection. In addition to its direct impact on the tumor microenvironment, our research builds on recent evidence that bacterial virulence proteins can initiate in situ vaccination against tumors.^61^ We hypothesize that the tumor cell debris generated during T cell-mediated tumor destruction, combined with the adjuvant properties of *E. coli* MG1655, could serve as a potent in situ tumor vaccine. This vaccine effect may not only target the primary tumor but could also inhibit the growth of distant tumors or metastases, potentially providing long-term protection against cancer.

The potential benefits of this strategy extend beyond its direct impact on the primary tumor. By broadening the targeted antigen pool, this approach could educate endogenous T cells to recognize and eliminate multiple tumor-associated antigens. This could lead to a more comprehensive immune response against cancer, further enhancing the effectiveness of immunotherapy. As we integrated dual living therapies into our designed approaches, we observed that intratumorally injected *E. coli* MG1655 effectively killed tumor cells from within, while adoptively transferred T cells attacked tumor cells from the periphery toward inner tissues. This synergistic interplay led to the eradication of both murine melanoma and hepatocellular tumors. Notably, both the bacteria and the adoptively transferred T cells exhibit dynamic behavior in the body, capable of proliferation and achieving biological efficacy. Our study provides preliminary insights into the spatial dynamics of the interplay between these two therapeutic modalities. Looking ahead, various living therapeutic modalities, such as CAR-natural killer cell therapy, CAR-macrophage therapy, and oncolytic virus therapy, could be incorporated into this system for tumor treatment. A deeper understanding of the spatial and temporal dynamic interactions between these modalities will empower researchers to innovate and engineer more potent and patient-specific cancer treatments. In conclusion, our study offers a promising new approach to cancer treatment that harnesses the unique capabilities of bacteria to modify the tumor microenvironment and bolster the immune system’s response to cancer. By exploring the potential of in situ vaccination and broadening the targeted antigen pool, this strategy could lead to more effective and comprehensive immunotherapies for solid tumor treatments.

## Materials and methods

### Animals and study approvals

Our research complies with ethical regulations. Local Ethics Committees, Zhejiang Provincial Committee for Laboratory Animals (ZJCLA), approved the study protocol (ZJCLA-IACUC-20010318). C57BL/6 and OT-I mice were purchased from Charles River (China) and Hangzhou Medical College (China), respectively. Anti-hCD19 murine CAR transgenic mice, C57BL/6 background, were built by Prof. Dan Chen (East China Normal University). All mice used were female, aged 6–8 weeks, and housed in micro isolator cages with five mice in every cage.

### Cell line

All bacteria strain used in this work was *E. coli* MG1655 purchased from BeNa Culture Collection (BNCC361739). The B16F10-OVA was a general gift from Prof. Xiao Zhao (CAS Key Laboratory for Biomedical Effects of Nanomaterials and Nanosafety & CAS Center for Excellence in Nanoscience, National Center for Nanoscience and Technology). Panc02 cells were bought from Pricella Life Science & Technology Co., Ltd. B16F10-OVA-luci and Panc02-hCD19-luci cells were constructed through lentivirus transfection method. Hepa1-6-OVA, B16F10-OVA cells, B16F10-OVA-luci, and Panc02-hCD19-luci were cultured in Dulbecco’s modified Eagle’s medium (DMEM) with 10% fetal bovine serum (FBS) (Gibco) and 1% penicillin/streptomycin (Solarbio).

### Antibodies

The fluorescent antibodies for flow cytometry analysis include CD45 (Biolegend catalog no. 103108, FITC-conjugated), CD19 (Biolegend catalog no. 363006, APC-conjugated), CD3 (Biolegend catalog no. 100206, PE-conjugated), CD4 (Biolegend catalog no. 100412, APC-conjugated), CD8a (Biolegend catalog no. 100752, BV510-conjugated), CCR5 (Biolegend catalog no. 107011, APC-conjugated), CD69 (Biolegend catalog no. 104512, PE/Cy7-conjugated), CD11b (Biolegend catalog no.101263, BV510-conjugated), F4/80 (Biolegend catalog no. 123110, PE-conjugated), CD206 (Biolegend catalog no. 141720, PE/Cy7-conjugated), CD11c (Biolegend catalog no. 117308, PE-conjugated), I-A/I-E (Biolegend catalog no. 107636, BV510-conjugated), CD80 (Biolegend catalog no. 104714, APC-conjugated), CD86 (BD Pharmingen, catalog no. 2403262, PerCP-eFluor 710-conjugated), H-2Kb bound to SIINFEKL (Biolegend catalog no. 141607, PE/Cy7-conjugated), CD16/32 TruStain FcX (BioLegend, catalog no. 156603/156604). Depending on their brightness, the antibodies were diluted 100 times during staining.

### Luminex cytokine determination assay

Briefly, when B16F10-OVA tumor grew to ~300 mm^3^, different therapeutic formulations were adopted (i.t. *E. coli* dosage 5 × 10^7^; i.v. OT-I T cell dosage 2 × 10^6^). On the third day, tumors were resected, homogenized (buffer: 150 mM NaCl, 20 mM Tris–Cl, 1 mM EGTA, 1 mM EDTA, 1% Triton-X-100, pH 7.5), and centrifuged. The supernatant was sent for Luminex assay quantification following product instructions (BIO-RAD, 12009159). Data was analyzed by Milliplex Analyst (Version 5.1)

### Isolation of tumor-reactive T cells

For OT-I T cells, OT-I mice spleens were first derived, disrupted, and filtered through a 70-μm strainer. Red blood cells were lysed by red blood cell lysis buffer (Solarbio) for 5 min at room temperature. The derived splenocytes were washed twice with 1 × PBS (500 × *g*, 3 min) and resuspended into 25 mL complete RPMI medium supplemented with 1% *v*/*v* sodium pyruvate (Gibco), 0.1% v/v 2-mercaptoethanol (Gibco), mouse IL-2 (10 ng mL^−1^, PeproTech), and OVA_257-264_ peptide (1 μM, Invivogen) for three days per spleen. The acquired cells were tested by flow cytometry (CytoFlex S, Beckman Coulter). The purity of CD8 T cells can reach ~90% (Supplementary Fig. 3). For the isolation of murine anti-hCD19 CAR-T cells, the spleens of CAR transgenic mice were harvested, disrupted, and filtered through a 70-μm strainer. Red blood cells were lysed by red blood cell lysis buffer (Solarbio) for 5 min at room temperature. Anti-hCD19 CD8 T cells were isolated by the EasySep Mouse CD8 T Cell Isolation Kit (Stem Cell) according to the protocol. A total of 2 × 10^7^ CD8 T cells were resuspended into 5 µg CD3 (Biolegend) and 5 µg CD28 (Biolegend) antibody-precoated T75 flask with 25 mL complete RPMI medium supplemented with 1% v/v sodium pyruvate (Gibco), 0.1% *v*/*v* 2-mercaptoethanol (Gibco), mouse IL-2 (10 ng mL^−1^, PeproTech) for two-day activation before use.

### In vitro tumor cell killing assay

B16F10-OVA-luci tumor cells (1 × 10^4^) were placed per well in a 96-well plate. Different amounts of OT-I CD8 T cells were added into the system at an effector: target (E: T) ratio of 8/1 to 1/8 and 0 and further co-cultured for 24 h. D-luciferin (100 μL, 1.5 mg mL^−1^) was added to each well, and the bioluminescence intensity was detected by the microplate reader (Thermo Scientific). Tumor cell killing efficiency was determined through calculation = (Intensity_ctrl_−Intensity_experiment_)/ Intensity_ctrl_ × 100%. The therapeutic efficacy of anti-hCD19 CAR-T cells against Panc02-hCD19-luci and TA99 CAR-T cells against B16-luci were tested similarly.

### In vitro transwell migration assay of T cells

Mice bearing ~150 mm^3^ B16F10-OVA tumors were treated with either i.t. 20 μL PBS or i.t. 20 μL 5 × 10^7^ CFU *E. coli* MG1655. On the second day, tumor tissues were resected from mice and directly ground over 70-μm strainer and using complete DMEM culture supplemented with 50 μg mL^−1^ gentamycin (G432053, Solarbio) to dilute cell density to 5 × 10^5^ per mL and centrifuged at 10000×*g* for 30 min to obtain the tumor interstitial fluid. 2 × 10^5^ B16F10-OVA cells with the 400 μL diluted tumor interstitial fluid were placed at the bottom well and 5 × 10^5^ isolated T cells in 200 μL were placed on the upper well (5 μm, Corning) and cultured for 24 h. The bottom well was subjected to IVIS (PerkinElmer, USA) to determine the bottom-well intensity of CFSE signals. All the tumor cells and OT-I T cells located at the bottom well were harvested and added with 10 μL counting beads (BioLegend) and were tested by flow cytometry (CytoFlex S, Beckman Coulter) to determine the migration quantity of T cells. OT-I T cells to beads ratio was analyzed by calculation = FITC^+^APC^−^ fraction/FITC^−^APC^+^ fraction. Data was analyzed by Flowjo (10.8.1).

### Impact of TAM depletion or CCL5 neutralization on OT-I T cell infiltration

OT-I T cells were stained by cell tracker deep red (Invitrogen) in cold PBS buffer for thirty minutes. The treatment schedule was initiated upon tumor reached 80 mm^3^ (Day 8). Mice in the clodronate liposome group were intratumoral injected with 20 μL clodronate liposome (FormuMax, F70101C-A-2). The next day (Day 9), each mice were intratumoral injected with 20 μL 5 × 10^7^ *E. coli* MG1655, 20 μg CCL5 antibody (+/–), and intravenously injected with 100 μL OT-I T cells. The next day, tumors were resected from mice and mechanically disrupted and filtered through a 70-μm strainer. Tumor-derived single-cell suspensions were pre-stained with corresponding antibodies and subjected to flow cytometry. Tumor inhibition experiment was repeated similarly.

### Flow cytometry analysis of immune cell phenotypes

For intratumor immune cell phenotype determination, after therapeutic drugs were applied for two days, the whole tumor tissues were resected and minced with scissors and further digested in RPMI 1640 medium containing 1 mg mL^−1^ collagenase Type IV (Gibco), 100 μg mL^−1^ DNase I (Sigma-Aldrich) at 37 °C with shaken for 1 h. These solutions were then filtered through 70 μm filters to obtain the single-cell suspensions. Following the removal of red blood cells (RBC) using the RBC lysis buffer, the cells were stained with fluorescence-labeled antibodies for 1 h and then washed twice with cell staining buffer for flow cytometric analysis. For the blood sample, 50 μL whole blood of each mouse was obtained in the heparin tube. After being treated with RBC lysis buffer (Solarbio) twice, cells were stained and washed as previously described for flow cytometric analysis. CytoFlex LX Flow Cytometer tested samples, and the data was analyzed by Flowjo (10.8.1).

### Intratumoral and serum cytokine assay

Briefly, C57BL/6 mice were inoculated with 1 × 10^6^ B16F10-OVA tumor cells. When the tumor volume reached about 150 mm^3^, 5 × 10^7^ CFU *E. coli* were intratumor injected. Whole tumor tissues and mice serum were collected on day 0 (before bacteria injection), day 1, 4, and 7 after bacteria injection. The whole tumor tissues were weighed, added with 600 μL 1 × PBS, mechanically cut into small pieces, and centrifuged at 500×*g* for 5 min to obtain the supernatant. The supernatant and serum were quantified with TNF-*α* (EK282/4), IFN-*γ* (EK280-3), IL-1*β* (EK201/B), IL-6 (EK206/3), IL-10 (EK210/4) concentration by ELISA kit (MultiSciences).

### Statistical analysis

All statistical analysis was performed by GraphPad Prism 9 software. The difference between groups was analyzed by Student’s *t*-test. Multiple group comparison was analyzed by One-way ANOVA followed by Tukey post-hoc analysis. Significance was considered if the *P* value was less than 0.05.

## Supplementary information

Supplementary Materials

## Data Availability

We declare that all data supporting the findings of this study are presented in the main text and supplementary information. The source data are available on Science data Bank (https://www.scidb.cn/s/n63I3q).
