# Supplementary material for: Remolding the tumor microenvironment by bacteria augments adoptive T cell therapy in advanced-stage solid tumors
Source: Signal Transduct Target Ther. 2024 Nov 22;9:307. doi: 10.1038/s41392-024-02028-3 (PMC11582571; doi:10.1038/s41392-024-02028-3)
Supplement: Supplementary file 1 — Supplementary Materials [file 41392_2024_2028_MOESM1_ESM.docx]

**Supplementary Materials for**

Remolding the tumor microenvironment by bacteria augments adoptive T cell therapy in advanced-stage solid tumors

Chaojie Zhu^1,2,3^†, Chao Liu^4^†, Qing Wu^2,3^†, Tao Sheng^2^, Ruyi Zhou^2^, En Ren^2,3^, Ruizhe Zhang^2,3^, Zhengjie

Zhao^2^, Jiaqi Shi^2,3^, Xinyuan Shen^2,3^, Zhongquan Sun^1^, Zhengwei Mao^5^, Kaixin He^2^, Lingxiao Zhang^6^,

Yuan Ding^1,7,8^*, Zhen Gu^2,3,9,10^*, Weilin Wang^1,7,8^*, Hongjun Li^1,2,3^*

Correspondence to: [hongjun@zju.edu.cn](mailto:hongjun@zju.edu.cn) (H.L.); [wam@zju.edu.cn](mailto:wam@zju.edu.cn) (W.W.); [guzhen@zju.edu.cn](mailto:guzhen@zju.edu.cn) (Z.G.); [dingyuan@zju.edu.cn](mailto:dingyuan@zju.edu.cn) (Y.D.)

**This PDF file includes:**

Materials and Methods

Figures. S1 to S23

Materials and Methods

**Immunofluorescence staining**

Embedded tissue samples were sectioned at 10 μm. The slides were first deparaffinized, then rehydrated, and heated for antigen retrieval in sodium citrate buffer solution (pH = 6.0). The tumor sections were incubated at 4 °C with primary antibodies overnight. Fluorescent secondary antibodies were applied for 1 h at room temperature. Primary antibodies were CD31 (Abcam, ab182981, 1: 2000), Ki67 (CST, 9129, 1: 400), CD3 (ZSGB-Bio, ZA-0503). TUNEL staining was conducted according to the products’ instructions (Nanjing Vazyme Biotech Co, A113-03). *E. coli* was stained by fluorescence *in situ* hybridization (GENER Bio, EUB338-FITC, A316706). Briefly, the tumor section after being stained with CD3 was added with proteinase K at 37 °C for 30 minutes. After tumor sections were soaked in pre-hybrid buffer at 42 °C for two hours, 100 µM Hybrid probe was diluted (final concentration 1 µM) to react with the sample at 42 °C for 18 hours. The slides were scanned by Pannoramic SCAN Ⅱ (3DHISTECH Ltd.)

**T cell absorption toward tumor tissue co-culture assay**

Mice bearing ~ 150 mm^3^ B16F10-OVA tumors were treated with either *i.t.* 20 μL PBS or *i.t.* 20 μL 5 × 10^7^ CFU *E. coli* MG1655. On the second day, tumor tissues were resected from mice and co-cultured with 5 × 10^5^ Cy5-labeled OT-I T cells in complete DMEM culture supplemented with 50 μg mL^−1^ gentamycin (G432053, Solarbio) for 24 hours. The Cy5 signal intensity of tumor tissues were tested by IVIS (PerkinElmer, USA).

**T cell infiltration toward tumor inner tissue co-culture assay**

Mice bearing ~ 150 mm^3^ B16F10-OVA tumors were treated with either *i.t.* 20 μL PBS or *i.t.* 20 μL 5 × 10^7^ CFU *E. coli* MG1655. On the second day, tumor tissues were resected from mice, cut into half pieces, and co-cultured with 5 × 10^5^ Cy5.5-labeled OT-I T cells in the environment of complete DMEM culture supplemented with 50 μg mL^−1^ gentamycin for 24 hours. The Cy5.5 signal intensity of tumor tissues was determined by IVIS (PerkinElmer, USA).

**Quantification of T cell infiltration into tumor tissue**

Isolated OT-I T cells were stained with Cy5-NHS ester in cold PBS for 30 minutes, washed with fresh PBS twice, and diluted to 2 × 10^7^ mL^−1^. Mice bearing ~ 150 mm^3^ B16F10-OVA tumors were treated with either *i.t.* 20 μL PBS or *i.t.* 20 μL 5 × 10^7^ CFU *E. coli* MG1655. All mice were intravenously injected with 100 μL OT-I T cells. Mice were tested by IVIS (PerkinElmer, USA) to determine the Cy5 intensities of the tumor area every two hours. On the second day, tumor Cy5 intensities were determined by IVIS. To subject tumor tissues for flow cytometry examination, OT-I T cells were stained by cell tracker deep red (Invitrogen) in cold PBS buffer for thirty minutes. The treatment schedule was the same as the above. The next day, tumors were resected from mice and mechanically disrupted and filtered through a 70-μm strainer. Tumor-derived single-cell suspensions were pre-stained with corresponding antibodies and subjected to flow cytometry.

**Co-culture of *E. coli* with BMDM**

Briefly, 1 × 10^5^ BMDM were cultured in RPMI1640 complete medium and placed at the bottom well of 12-well plate. Four hours later, 1 × 10^7^ *E. coli* MG1655 were added into the indicated wells and co-cultured for 16 hours. The cell culture supernatant was harvested and tested by ELISA Kit (CCL5, MultiSciences, EK2129). The left supernatant was kept for transwell migration assay. And the BMDM was harvested and stained for flowcytometry analysis.

**CCL5 blockade transwell migration assay**

Briefly, 1× 10^5^ isolated OT-I T cells in 200 μL RPMI1640 complete medium were added into the upper well. The bottom wells were added with indicated pre-treated medium (600 μL). CCL5 neutralization antibody (R&D system, MAB478) were added at 2 μg per well. 16 hours later, the cells at bottom wells were harvested, stained with anti-CD8 antibodies, added with 10 μL counting beads (Biolegend) and were tested by flow cytometry (CytoFlex S, Beckman Coulter) to determine the migration quantity of T cells.

**Preparation of TA99 murine CAR-T cells**

Murine CAR (Chimeric Antigen Receptor)-expressing constructs were generated and utilized to produce mouse CAR-T cells, following previous reported protocols (*Nat. Immunol. 2021*, *22*, 746-756.; *Science 2019*, *365*, 162–168.). For viral vector production, Phoenix-Eco cells were transfected with a TA99 CAR-encoding plasmid employing the calcium phosphate transfection method. Subsequently, the medium containing calcium phosphate precipitates was exchanged for fresh, antibiotic-free complete medium. Virus-laden supernatant was harvested at 48- and 72-hours post-transfection.

Splenocytes isolated from wild-type (WT) mice were activated using mouse T-activator CD3/CD28 Dynabeads (Invitrogen/Thermo Fisher Scientific) in a T-cell culture medium fortified with IL-2 (10 ng mL^−1^) and IL-7 (10 ng mL^−1^), maintained at a cell density of 2 × 10^6^ cells mL^−1^. After 24 to 48 hours of stimulation, splenocytes were purified *via* Ficoll-Paque Plus density gradient centrifugation.

For the generation of mouse CAR-T cells, the virus-containing supernatant was added to 6-well plates pre-coated with 10 μg mL^−1^ protamine (Sigma-Aldrich, 194729). Spinoculation was performed by centrifugation at 2,000 g for 2 hours at 32°C to facilitate viral transduction. Subsequently, activated T cells were added to the wells at a density of 1 × 10^6^ cells mL^−1^, followed by a second centrifugation step at 400 × g for 30 minutes at 32 °C to enhance cell-virus interaction. The cells were then incubated in a cell incubator.

Post-transduction, the T cells were cultured overnight in fresh complete medium supplemented with mouse IL-2 (20 ng mL^−1^) to support their growth and CAR expression. The transduction efficiency of the CAR-T cells was assessed 24 hours later via flow cytometry, utilizing DAPI (Biolegend, 422801) for viability staining and PE Conjugated Myc-Tag (9B11) Mouse mAb (CST, 3739) to detect CAR expression. For expansion and subsequent applications, the mouse CAR-T cells were maintained at a density of 2 × 10^6^ cells mL^−1^. These cells were ready for adoptive transfer into experimental models or for in vitro functional assays 48 hours post-transduction.

**Therapeutic efficacy in mice small melanoma tumor model**

The mice melanoma tumor model was established by subcutaneously injecting 1 × 10^6^ B16F10-OVA cells per C57BL/6 mice. When tumor volume reached about 50 mm^3^, mice were assigned into different groups and were first intratumoral injected with 20 μL PBS or 20 μL 5 × 10^7^ CFU *E. coli* MG1655. And mice were intravenously injected with 100 μL PBS or 100 μL 2 × 10^6^ OT-I T cells. The size of the tumor was monitored by the equation (Volume = Length × Width^2^ / 2).

**Therapeutic efficacy in subcutaneous pancreatic tumor model**

The mice subcutaneous pancreatic carcinoma model was established by subcutaneous injection of 8 × 10^5^ Panc02-hCD19-luci cells into C57BL/6 mice. When tumor volume reached about 150 mm^3^, mice were assigned into different groups and were first intratumor injected with 20 μL PBS or 20 μL 5 × 10^7^ CFU *E. coli* MG1655. And mice were intravenously injected with 100 μL PBS or 100 μL 2 × 10^6^ anti-hCD19 murine CAR-T cells. The size of the tumor was monitored by the equation (Volume = Length × Width^2^ / 2).

**Therapeutic efficacy in the bilateral melanoma tumor model**

The mice bilateral melanoma tumor model was established by subcutaneous injecting of 5 × 10^5^ B16F10-OVA cells (each flank) into C57BL/6 mice at double flanks. On day 8, the larger tumor was chosen as the primary tumor, and the smaller one was selected as the distal tumor for each mouse. The larger tumor of each mouse was selected as the primary tumor and was intratumor injected with 20 μL PBS or 20 μL 5 × 10^7^ CFU *E. coli* MG1655. And mice were intravenously injected with 100 μL PBS or 100 μL 2 × 10^6^ OT-I T cells. The size of the tumor was monitored by the equation (Volume = Length × Width^2^ / 2).

**Therapeutic efficacy in large melanoma tumor model**

The mice melanoma tumor model was established by subcutaneous injection of 1 × 10^6^ B16F10-OVA cells into C57BL/6 mice. When tumor volume reached about 400 mm^3^, mice were assigned into different groups and were first intratumoral injected with 50 μL PBS or 50 μL 5 × 10^7^ CFU *E. coli* MG1655. And mice were intravenously injected with 100 μL PBS or 100 μL 2 × 10^6^ OT-I T cells. The size of the tumor was monitored by the equation (Volume = Length × Width^2^ / 2). For B16-luci tumor model, 1 × 10^6^ B16-luci cells were subcutaneously injected into C57BL/6 mice. When the tumor volume reached 300 mm^3^, mice were assigned into different groups and were first intratumoral injected with 50 μL PBS or 50 μL 5 × 10^7^ CFU *E. coli* MG1655. And mice were intravenously injected with 100 μL PBS or 100 μL 2 × 10^6^ TA99 murine CAR-T cells. Mice were monitored for body weight, temperature, and toxic score. The standard of toxic score is referred to *Cell Rep Med. 2023;4(9):101161.*

**Therapeutic efficacy in subcutaneous hepatocellular carcinoma tumor model**

The mice subcutaneous hepatocellular carcinoma model was established by subcutaneous injection of 2 × 10^5^ Hepa1-6-OVA cells into C57BL/6 mice. When tumor volume reached about 400 mm^3^, mice were assigned into different groups and were first intratumor injected with 50 μL PBS or 50 μL 5 × 10^7^ CFU *E. coli* MG1655. And mice were intravenously injected with 100 μL PBS or 100 μL 2 × 10^6^ OT-I T cells. The size of the tumor was monitored by the equation (Volume = Length × Width^2^ / 2).

***E. coli* tumor colonization**

LuxCDABE-transduced *E. coli* was a kind gift from Dr. Mengchi Sun (School of Pharmacy, Shenyang Pharmaceutical University). Briefly, 5 × 10^7^ CFU *E. coli* were intratumor injected. Mice were subjected to IVIS (PerkinElmer, USA) to determine the bioluminescence intensities of the tumor area at the indicated time points.

**Routine blood and serological tests**

To assure the biosafety of this therapeutic modality, we retrieved whole blood from mice orbit two-day or one month after treatment. Serum was obtained by centrifuging whole blood in the anti-coagulant tube at 3000 × g for 30 minutes. Samples were sent to Hangzhou Liangying Technology Co., Ltd. (China) for analysis.


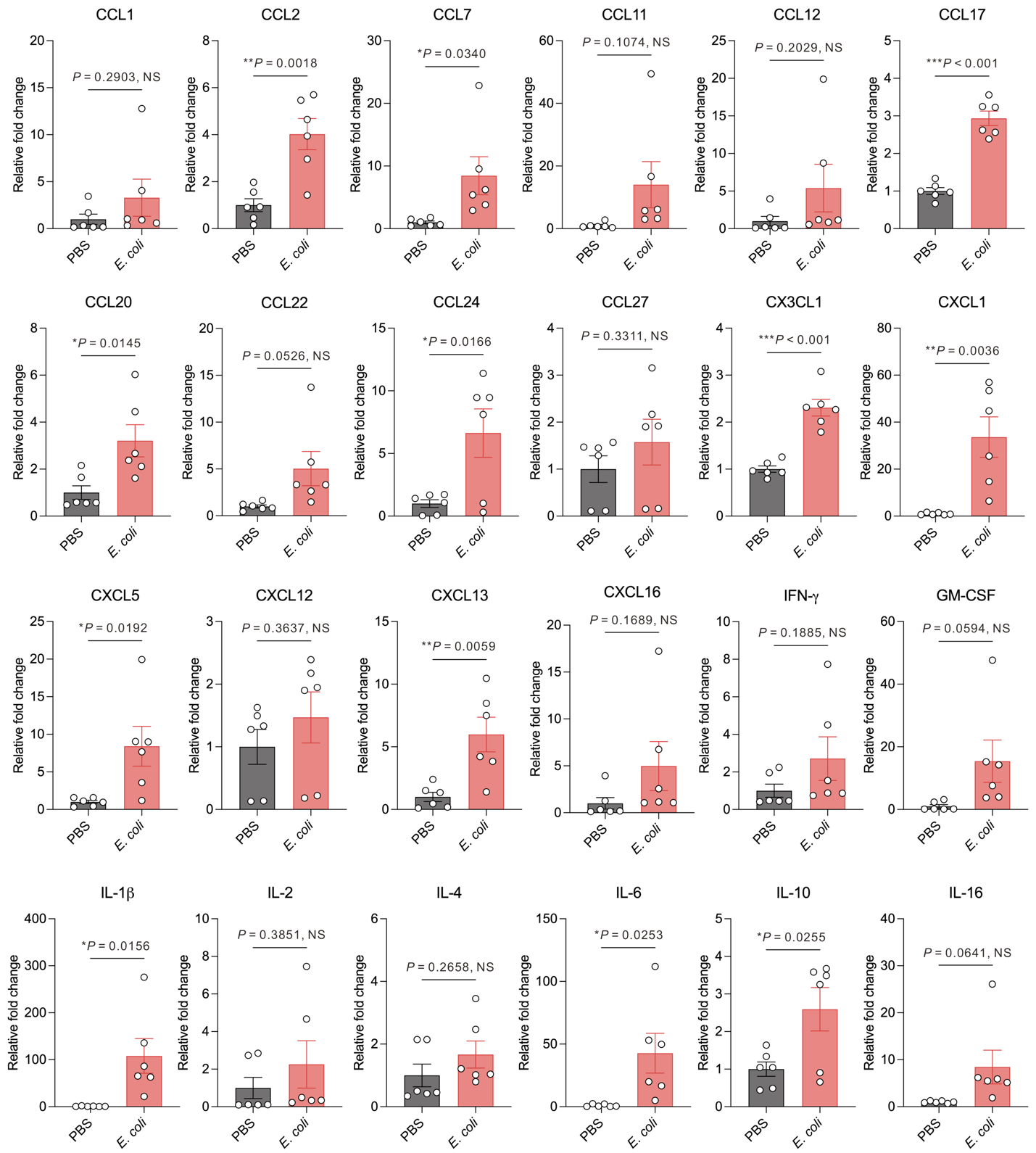


Figure. S1. Luminex determination of intratumor cytokine and chemokine content in the *E. coli* group compared to the PBS group. Data are shown as mean ± *s.e.m*., (*n* = 6 biologically independent animals). *P* values were determined by unpaired, two-tailed *t*-test. NS, no significance.


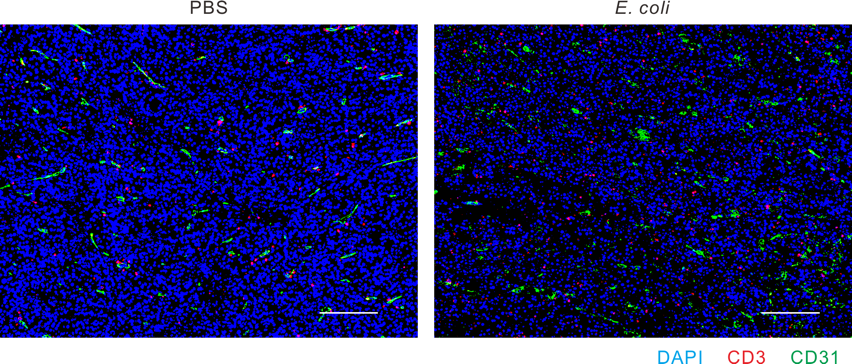


Figure. S2. Fluorescence staining of the intratumor CD31 and CD3 T cells, scale bar = 200 μm.


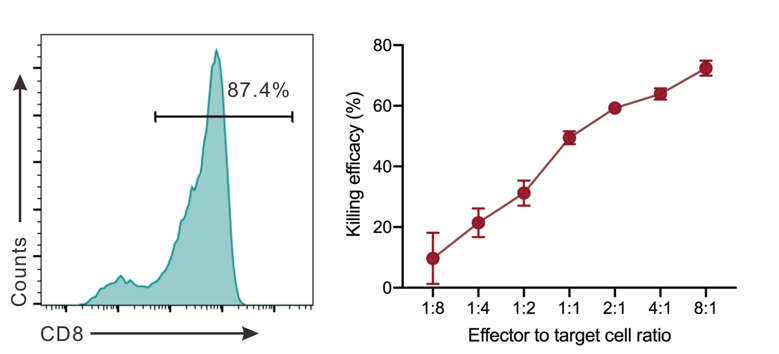


Figure. S3. Flow cytometry analysis of the CD8 T cell proportion of the derived OT-I T cells (left). Killing efficacy of OT-I T cells toward B16F10-OVA-luci tumor cells at different effector-to-target cell ratios (right), *n* = 3. Data are shown as mean ± *s.d.*.


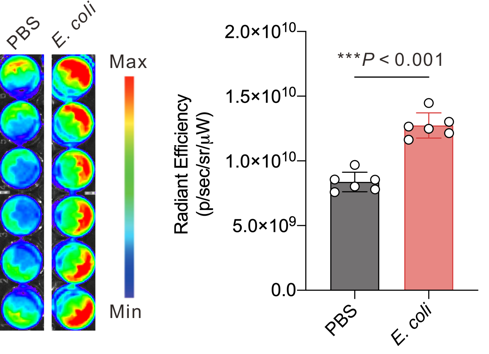


Figure. S4. IVIS image and statistical analysis of CFSE fluorescence signals of OT-I T cells in the bottom wells (*n* = 6). Data are shown as mean ± *s.d.*. *P* values were determined by unpaired, two-tailed *t*-test.


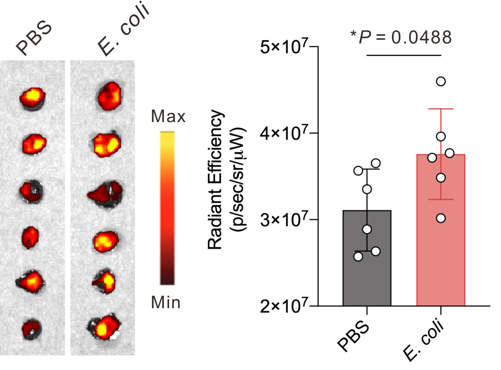


Figure. S5. IVIS image and statistical analysis of the half tumor and OT-I T cell (labeled by Cy5.5) co-culture infiltration assay (*n* = 6). Data are shown as mean ± *s.d.*. *P* values were determined by unpaired, two-tailed *t*-test.


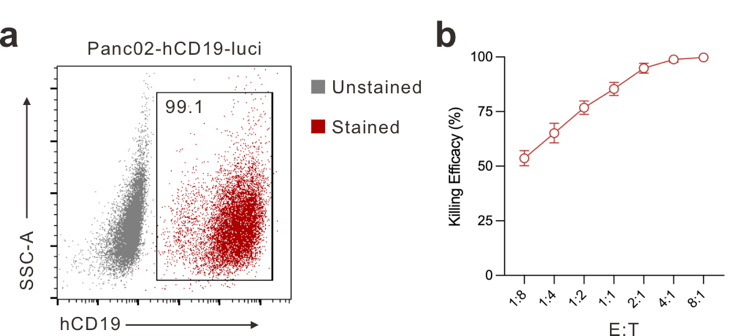


Figure. S6. a Flow cytometry analysis of the hCD19 positive proportion in the transduced Panc02 cells. Experiments were repeated three times with similar results. b Killing efficacy of anti-hCD19 CAR-T cells against Panc02-hCD19-luci (E: T = Effector cell: Target cell; *n* = 8 independent samples). CAR-T cells and tumor cells were co-cultured for 48 hours. Data are shown as mean ± *s.d.*.


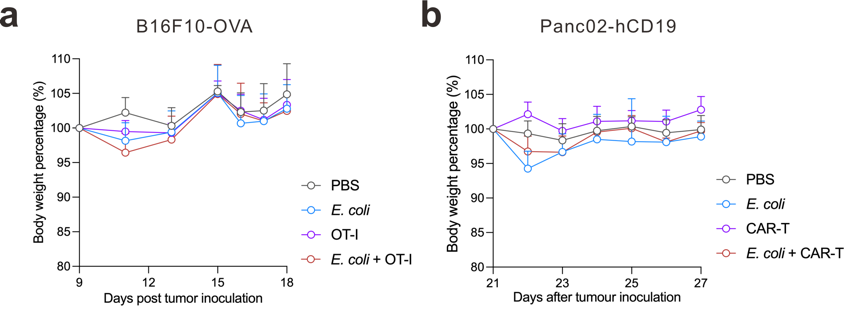


Figure. S7. a Body weight monitoring of different treatment groups in the B16F10-OVA tumor model (*n* = 6-8 biologically independent animals). b Body weight monitoring of different treatment groups in the Panc02-hCD19 tumor model (*n* = 5 biologically independent animals). Data are shown as mean ± *s.d.*.


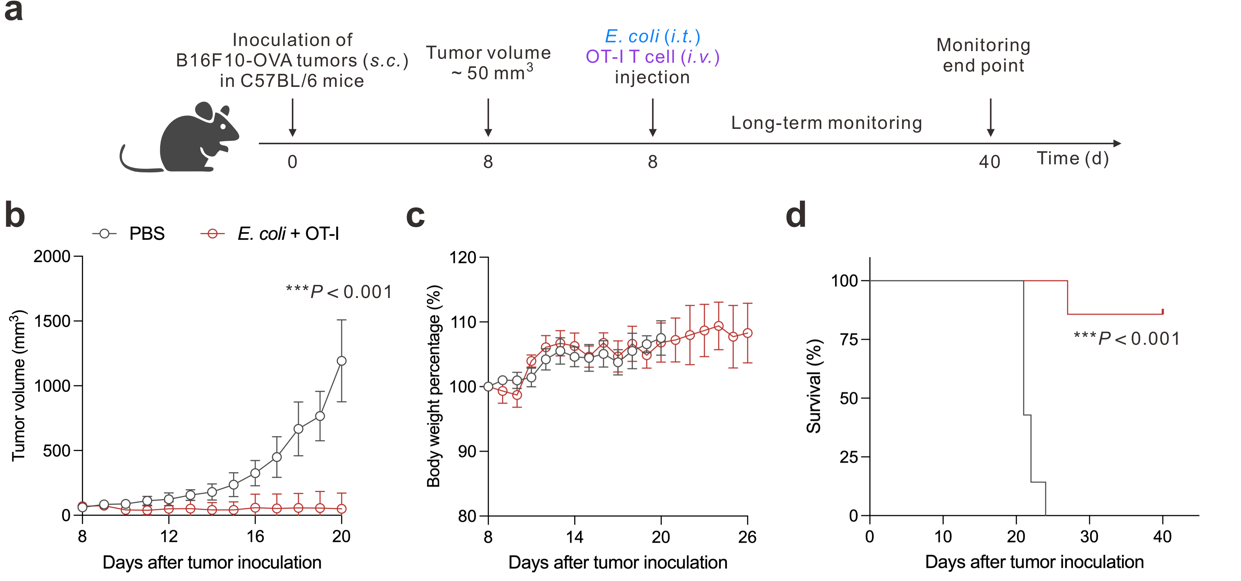


Figure. S8. Long-term efficacy of *E. coli* + OT-I in the B16F10-OVA tumor model. a Therapeutic schedule. b Tumor growth curves (*n* = 7 biologically independent animals). *P* values were determined by unpaired, two-tailed *t*-test. Data are shown as mean ± *s.d.*. c Body weight curves (*n* = 7 biologically independent animals). d Long-term survival monitoring (*n* = 7 biologically independent animals). Data are shown as mean ± *s.d.*. The survival statistical significance was analyzed by log-rank (Mantel-Cox) test.


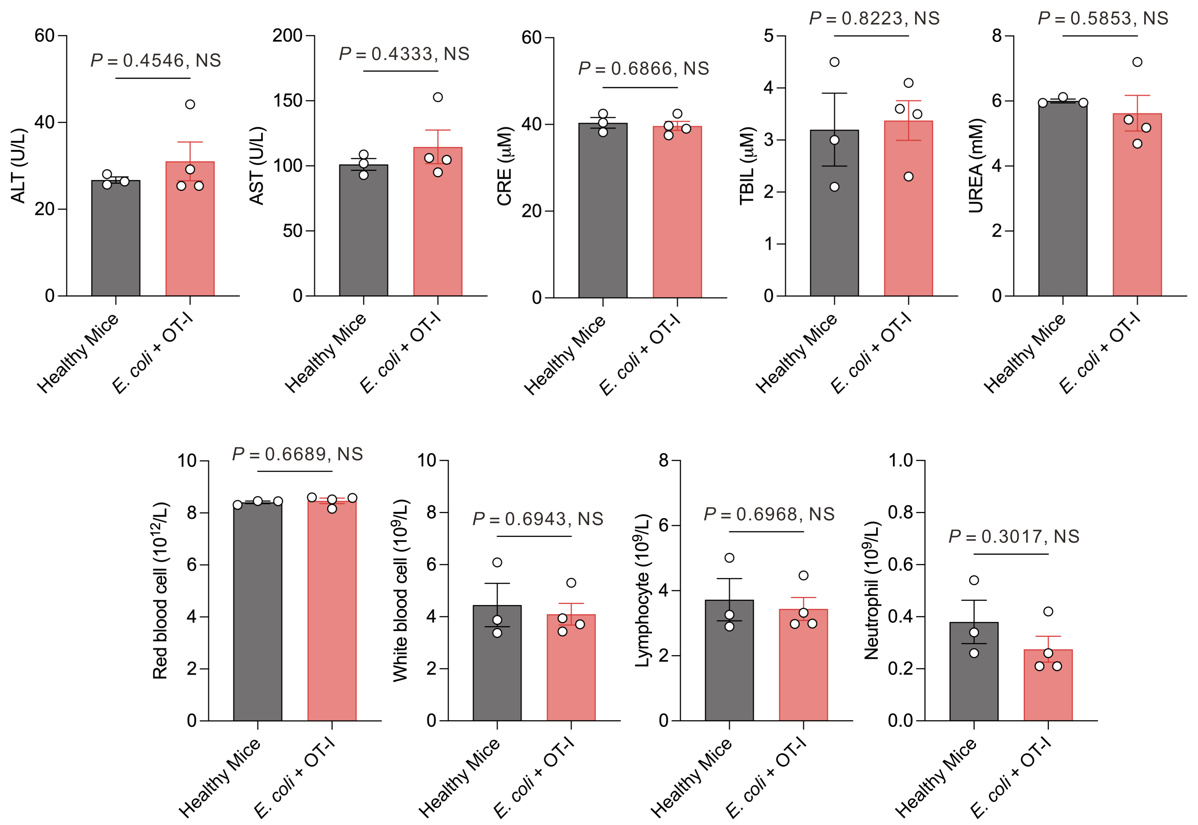


Figure. S9. Routine blood test and serological analysis of mouse from *E. coli* + OT-I group (*n* = 4 biologically independent animals). Same-aged healthy mice were taken as control group (*n* = 3 biologically independent animals). Data are shown as mean ± *s.e.m*.. *P* values were determined by unpaired, two-tailed *t*-test. NS, no significance. ALT: alanine aminotransferase; AST: aspartate aminotransferase; CRE: creatinine; TBIL: total bilirubin.


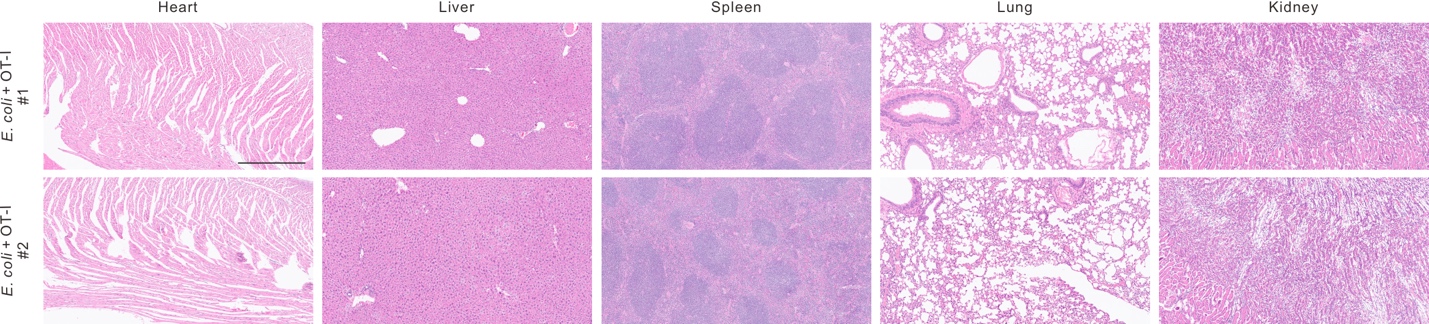


Figure. S10. H&E images of major organs from the *E*. *coli* + OT-I group (*n* = 2 biologically independent animals), scale bar = 500 μm.


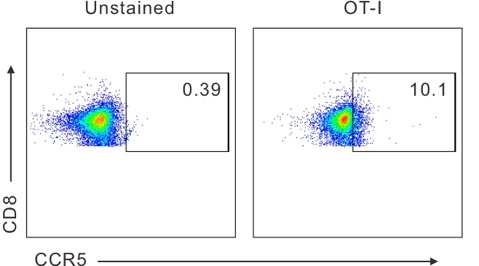


Figure. S11. Flow cytometry characterization of CCR5 expression on OT-I CD8 T cells.


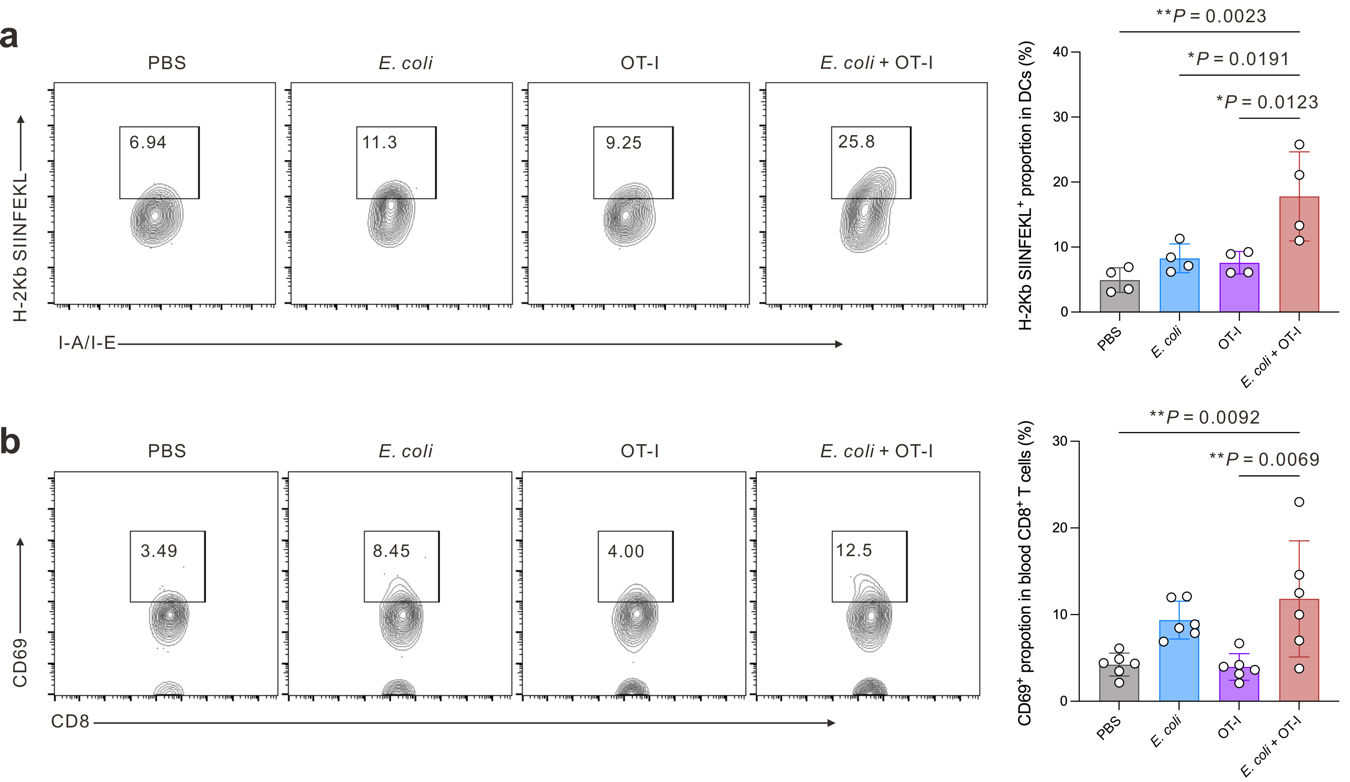


Figure. S12. Flow cytometry analysis of the abscopal immune effect. a Representative flow cytometry analysis of intratumor neoantigen-presenting dendritic cell proportions (*n* = 4 biologically independent animals). b Representative flow cytometry analysis of circulating activated CD8 T cell proportions (*n* = 6 biologically independent animals). Data are shown as mean ± *s*.*d*.. *P* values were determined by one-way ANOVA with a Tukey post hoc test.


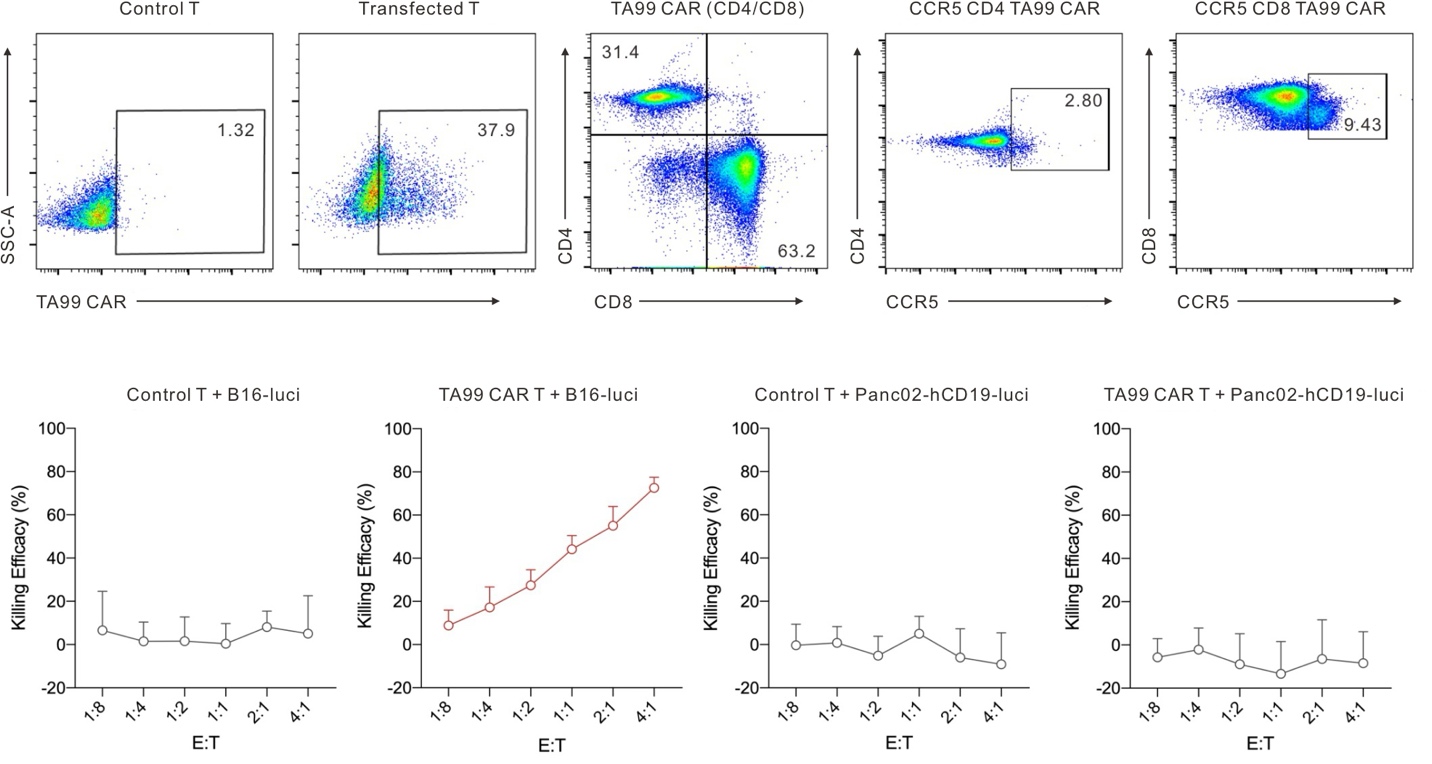


Figure. S13. Flow cytometry characterization of derived TA99 CAR-T cells (CAR positive ratio, CD4/CD8 ratio, and CCR5^+^ proportion). Tumor killing efficacy of transfected CAR-T cells and non-transfected T cells toward target cells B16-luci or non-target cells Panc02-hCD19-luci (*n* = 8 independent samples). Data are shown as mean ± *s.d*..


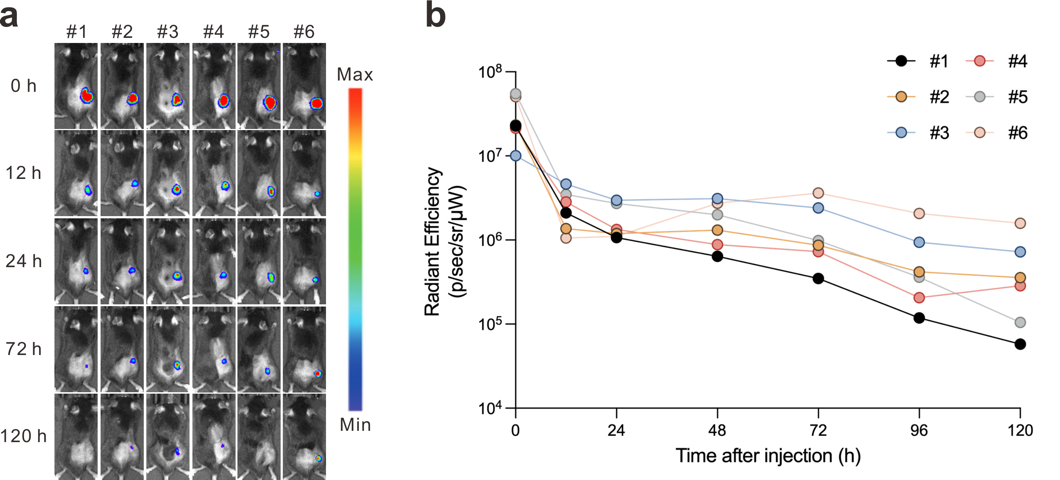


Figure. S14. a IVIS images of bioluminescence intensity of *E. coli*-LuxCDABE at different treatment time points. b Statistic diagram of the bioluminescence intensity changes over different time points (*n* = 6 biologically independent animals).


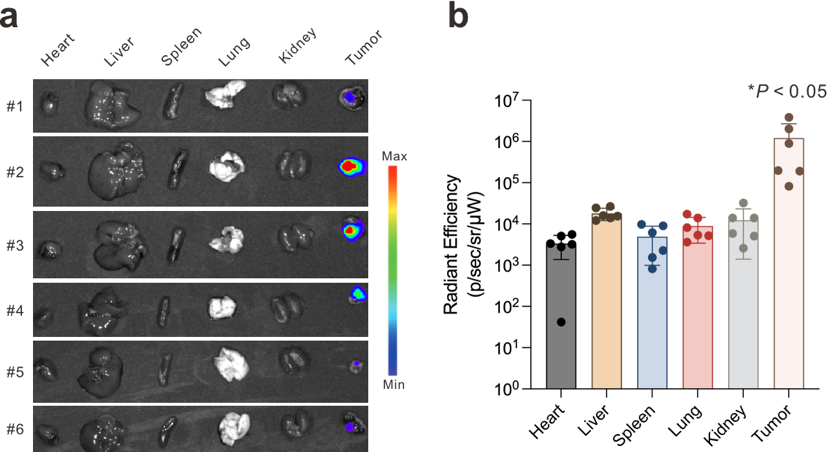


Figure. S15. a IVIS images of bioluminescence intensity of *E. coli*-LuxCDABE in major organs post 120 hours after treatment. b Statistic diagram of the bioluminescence intensities of different organs (*n* = 6 biologically independent animals). Data are shown as mean ± *s.d*.. *P* values were determined by one-way ANOVA with a Tukey post hoc test.


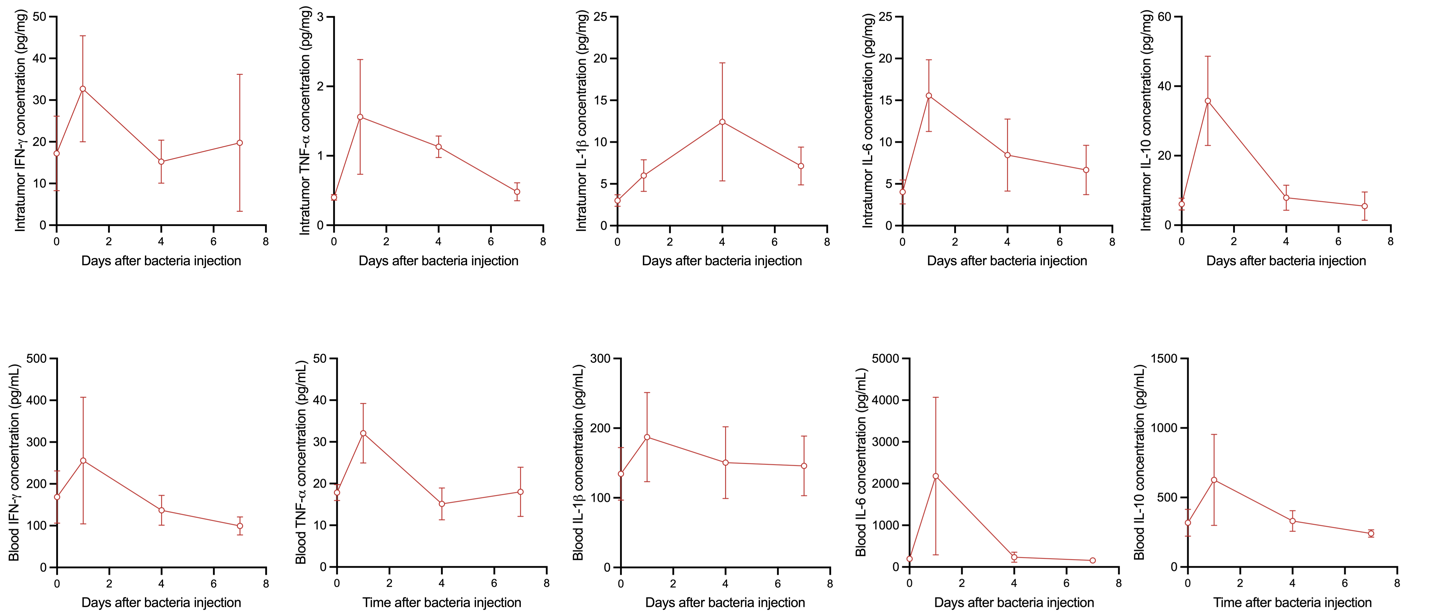


Figure. S16. ELISA assay of intratumor and serum cytokine levels (*n* = 4 biologically independent animals). Day 1 stands for one day after intratumoral bacteria injection. Data are shown as mean ± *s.d.*.


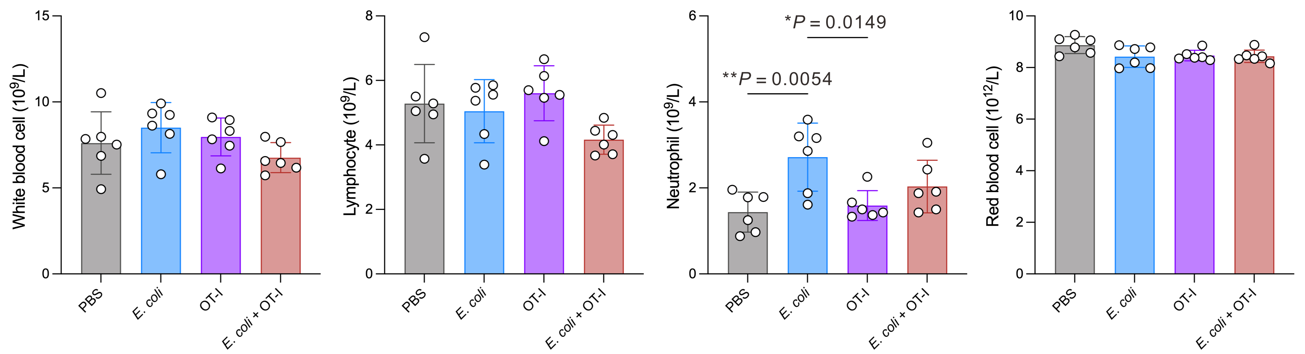


Figure. S17. Routine blood tests and statistical analysis of mice in different treatment groups (*n* = 6 biologically independent animals). Data are shown as mean ± *s.d.*. *P* values were determined by one-way ANOVA with a Tukey post hoc test.


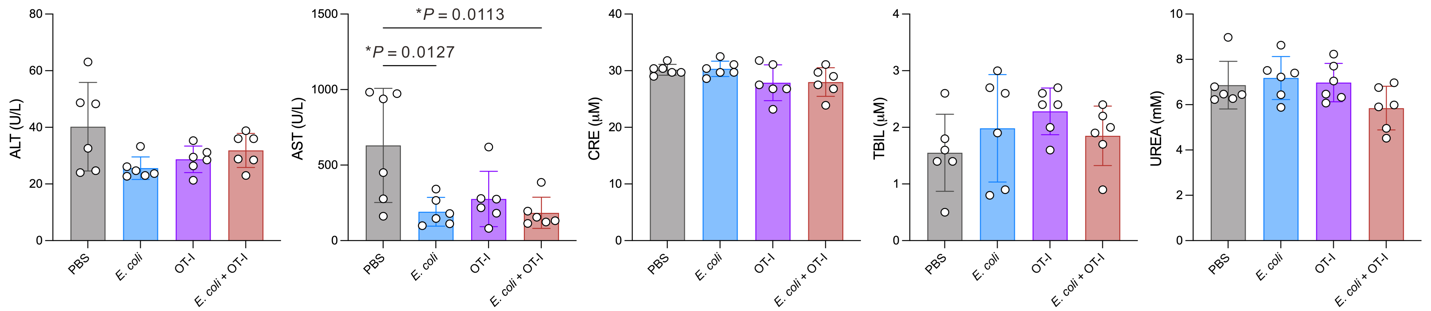


Figure. S18. Serological analysis of mice in different treatment groups (*n* = 6 biologically independent animals). ALT: alanine aminotransferase; AST: aspartate aminotransferase; CRE: creatinine; TBIL: total bilirubin. Data are shown as mean ± *s.d.*. *P* values were determined by one-way ANOVA with a Tukey post hoc test.


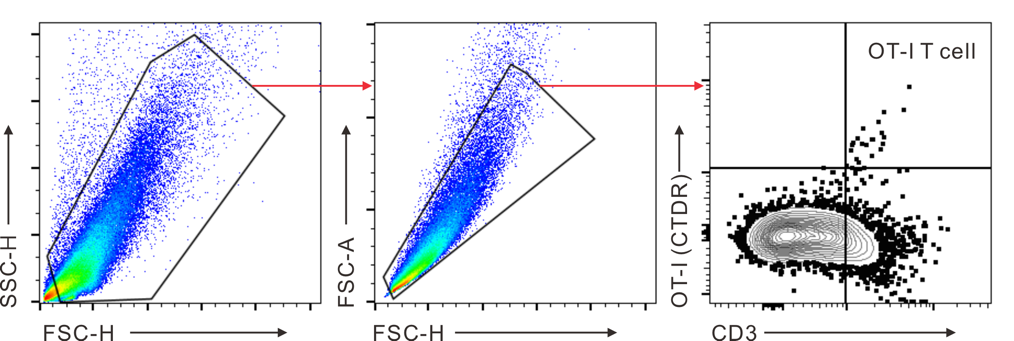


Figure. S19. Gating strategy of intratumor OT-I T cells.


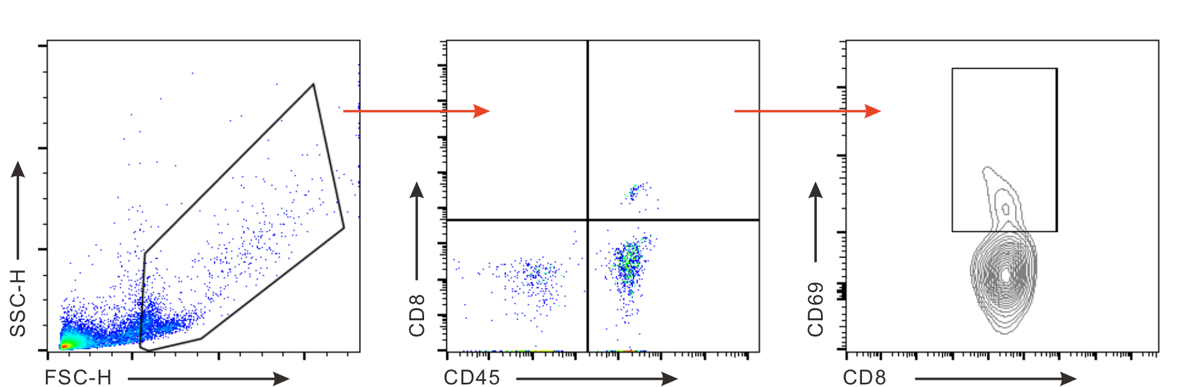


Figure. S20. Gating strategy of blood CD69 CD8 T cells.


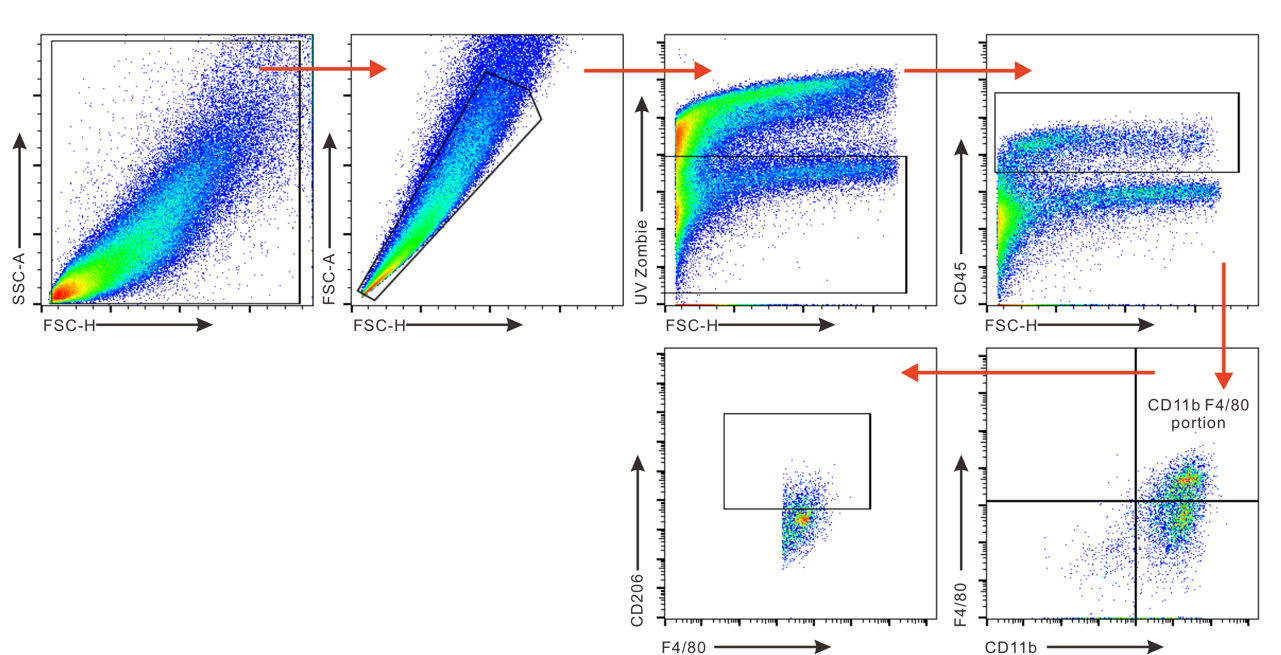


Figure. S21. Gating strategy of intratumor CD206 M2-like macrophages.


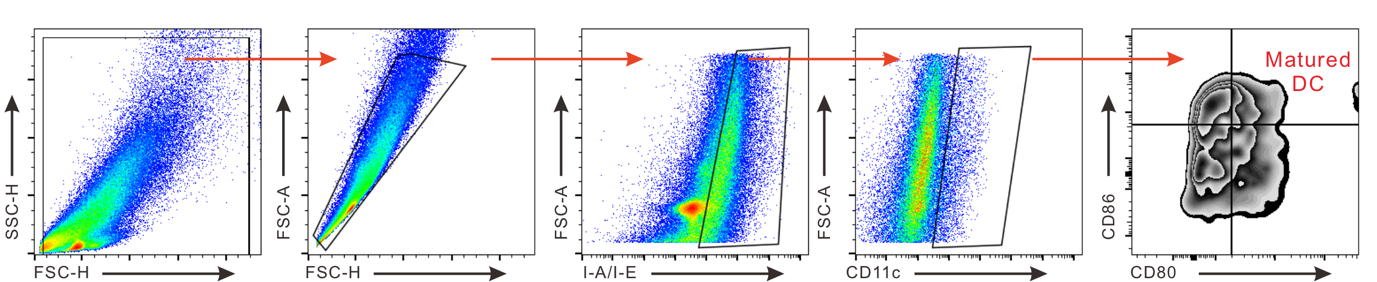


Figure. S22. Gating strategy of intratumor CD80 CD86 matured dendritic cells.


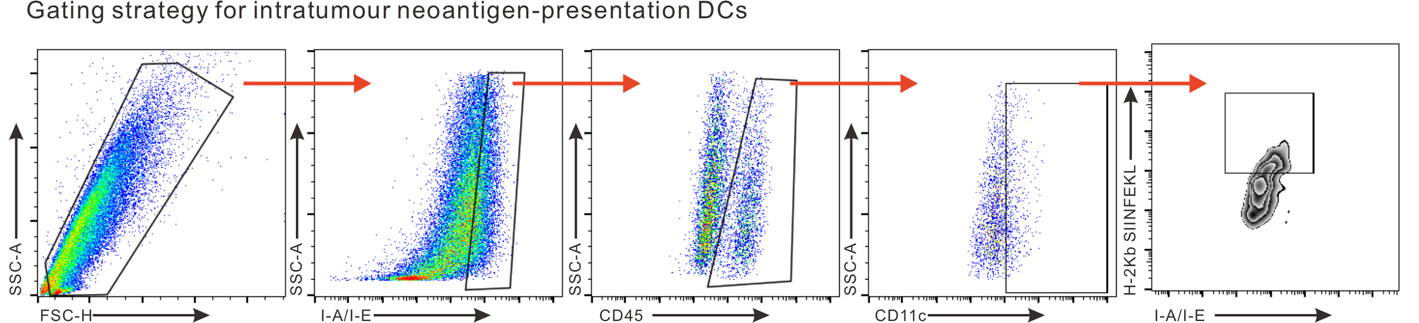


Figure. S23. Gating strategy of intratumor H-2Kb SIINFEKL dendritic cells.
